# PI3K is the dominant signalling pathway recruited by IRS proteins to mediate their effects on metabolism and growth

**DOI:** 10.1016/j.molmet.2026.102450

**Published:** 2026-10-06

**Authors:** Steven J. Millership, Nadia Tyler-Rubinstein, Elaine E. Irvine, Marika Charalambous, Agharul I. Choudhury, Santiago Vernia, Peter Voshol, Antonio Vidal-Puig, Lazaros C. Foukas, Dominic J. Withers

**Affiliations:** 1 Department of Metabolism, Digestion and Reproduction, Faculty of Medicine, Imperial College London, Du Cane Road, London, W12 0NN, UK; 2 MRC Laboratory of Medical Sciences, Du Cane Road, London, W12 0NN, UK; 3 Department of Medical and Molecular Genetics, Faculty of Life Sciences and Medicine, King's College London, London, SE1 9RT, UK; 4 The University of Cambridge Metabolic Research Laboratories, Wellcome Trust-MRC Institute of Metabolic Science, Cambridge, UK; 5 MRC Metabolic Diseases Unit, Wellcome Trust-MRC Institute of Metabolic Science, Addenbrooke's Hospital, Cambridge, CB2 0QQ, UK; 6 Institute of Healthy Ageing, Department of Genetics, Evolution and Environment, University College London, Gower Street, London, WC1E 6BT, UK; 7 Institute of Clinical Sciences, Faculty of Medicine, Imperial College London, Du Cane Road, London, W12 0NN, UK

**Keywords:** Insulin, IRS, PI3K, GRB2, Growth, Diabetes

## Abstract

**Objectives:**

Insulin and *Igf1* have pleiotropic effects on development, growth and metabolism. The activated insulin and IGF1 receptors engage insulin receptor substrate (IRS) proteins, intracellular adaptor molecules that recruit multiple downstream signalling pathways, including PI3K and GRB2-RAS-MAPK. Global deletion of IRS1 or IRS2 in mice results in divergent effects on growth, metabolism, and ageing. However, the precise signalling pathways recruited by IRS1 or IRS2 to mediate their physiological effects *in vivo* have not been defined.

**Methods:**

We generated knock-in mice in which either IRS1 or IRS2 was mutated to prevent downstream recruitment of PI3K or GRB2 and undertook phenotypic analysis of these animals.

**Results:**

Knock-in mice unable to recruit PI3K to IRS1 (*Irs1*^YnF^) or to IRS2 (*Irs2*^YnF^) had limited or no compensation in IRS2 and IRS1 signalling respectively. *Irs1*^YnF^ mice were growth restricted, with reduced adiposity and insulin resistance, but did not develop diabetes due to compensatory increase in β-cell mass. *Irs2*^YnF^ mice were obese and insulin resistant and developed diabetes due to β-cell failure. In contrast knock-in mice unable to recruit GRB2 to IRS1 (*Irs1*^Y891F^) or to IRS2 (*Irs2*^Y911F^) displayed normal growth and metabolism.

**Conclusions:**

PI3K recruitment by either IRS1 or IRS2 is the primary signalling mediator of the effects of these adaptor molecules on growth and metabolism in mice, and suggest that the other signalling pathways recruited by IRS1 and IRS2, including GRB2, have minimal or redundant roles.

## Highlights

• Knock-in IRS1 mice, unable to bind PI3K, phenocopy *Irs1* global null mice.
• Knock-in IRS2 mice, unable to bind PI3K, phenocopy *Irs2* global null mice.
• Mice unable to recruit GRB2 to either IRS1 or IRS2 display normal growth and metabolism.

## 1 Introduction

Insulin and insulin-like growth factor 1 (*Igf1*) have pleiotropic effects on development, growth and metabolism [[1], [2], [3], [4], [5]]. Alterations in their actions have been implicated in a wide range of diseases including diabetes, obesity, cancer and the ageing process [3,[6], [7], [8], [9]]. Insulin and IGF1 act through cell surface receptors with intrinsic tyrosine kinase activity, which in turn engage a complex, and only partially characterised, network of intracellular signalling molecules [1,4]. The best characterised targets of the activated insulin and IGF1 receptors are the insulin receptor substrate (IRS) proteins, which are intracellular adaptor molecules [[10], [11], [12]]. Once tyrosine phosphorylated on specific residues, IRS proteins can recruit multiple downstream signalling pathways, including phosphoinositide 3-kinase (PI3K) [13,14] and the GRB2-RAS-MAPK module [4], thereby permitting signal diversification, which is thought to underlie the complex actions of insulin and IGF1.

There are six IRS protein family members that all structurally contain N-terminal pleckstrin homology (PH) and phosphotyrosine binding (PTB) domains, which are involved in recruitment to activated receptors [10]. IRS protein C-terminal regions are of variable length and contain multiple tyrosine residues which are involved in downstream signalling molecule recruitment [15]. IRS1 and IRS2 are the major IRS proteins, widely expressed in most mammalian cells and tissues and have C-terminal regions capable of binding a diverse range of signalling molecules [10,[16], [17], [18]]. IRS3 is rodent-specific [10], IRS4 has restricted tissue expression [10], and IRS5/DOK4 and IRS6/DOK5 are less well characterised with limited evidence for significant roles in insulin action *in vivo* [19].

The signalling diversity downstream of IRS1 and IRS2 depends on the ability of their C-terminal regions (which only display 35% amino acid sequence identity to each other) to recruit a number of distinct proteins which bind to specific tyrosine phosphorylated structural motifs [[16], [17], [18]]. The best characterised binding module is the Src homology 2 (SH2) domain [20]. For example, the regulatory units of PI3K (p85α, p85β and p55γ) contain SH2 domains that bind to phosphorylated YxxM sequences and particularly YMxM sequences in the cytoplasmic tails of IRS1 and IRS2, leading to the activation of PI3K [15,17,18,21]. Similarly, GRB2, which links receptor tyrosine kinase receptors to the ERK cascade via activation of p21ras [22], binds to a specific YxNx motif in IRS1 and IRS2 [15,23]. Other signalling components recruited via SH2 domains to IRS proteins include the phosphatase SHP2 [15] and the kinases CSK [24], NCK [25] and CRK [26], thus demonstrating the potential for IRS proteins to recruit a wide range of signals. Furthermore, while the initial evidence for such interactions was derived from candidate studies, a phosphotyrosine interactome approach using SILAC and phosphotyrosine bait peptides combined with LC/MS has broadened the number of SH2-containing proteins that bind IRS1 and IRS2 and also identified proteins that bind independently of SH2 domains/phosphotyrosine interactions [18].

Global gene targeting of *Irs1* and *Irs2* in mice has revealed distinct but overlapping roles for these proteins in both physiology and disease processes [27]. Both genes have been independently deleted in mice by several groups using different targeting strategies and genetic backgrounds, but, in general, the following phenotypes have been consistently reported. *Irs1* knockout mice exhibit marked pre- and postnatal growth retardation, being about 40–45% smaller than control animals, but with relative preservation of brain size [[28], [29], [30]]. Metabolically, *Irs1* null mice are insulin resistant, primarily in skeletal muscle, but display only minimal changes in glucose tolerance due to compensatory hyperinsulinemia resulting from increased pancreatic β-cell mass [31,32]. Despite these metabolic abnormalities, *Irs1* null mice are long-lived and resistant to a range of age-related phenotypes [33,34]. *Irs2* knockout mice are equivalent in size to control animals in early life but with a marked reduction in brain size [30,31,35]. Metabolically, these mice display insulin resistance predominantly in the liver and adipose tissue and develop progressive diabetes due to a failure of β-cell compensation [31,32,35]. *Irs2* null mice also develop mild obesity due to hyperphagia, and female animals display infertility with pituitary, ovarian and reproductive hormone defects [36,37]. *Irs2* null mice are short-lived, primarily due to the development of diabetes [33].

While these studies provide strong evidence for critical roles for *Irs1* and *Irs2* in mammalian physiology and for their contribution to the pathophysiology of prevalent disease processes such as diabetes, obesity and the diseases of ageing, it remains unclear whether specific signalling pathways recruited by these proteins mediate specific physiological functions *in vivo*. The recruitment of multiple distinct signalling pathways perhaps suggests that a broad range of signalling mechanisms is required to mediate the physiological effects of insulin, but to date, there is limited experimental evidence to address this issue directly. An elegant series of *in vitro* reconstitution studies with *Irs1* examining the role of specific tyrosine residues in recruiting specific molecules have shown that PI3K binding mediates much of the mitogenic effects of insulin [38], with GRB2 binding largely unrequired for cell proliferation [23] and also that SHP2 binding acts to attenuate PI3K activity and protein synthesis [39]. However, these cell-based studies have not provided insights into potential physiological roles. *In vivo* studies globally deleting signalling pathways downstream of IRS proteins have also given limited insights either because they result in lethal or developmental phenotypes (e.g. *Grb2* [40]), have not directly affected insulin action (e.g. *Ship2* [41]) or, in the case of PI3K regulatory subunits, have resulted in complex and counterintuitive phenotypes [42]. We have previously shown that mice heterozygous for a kinase-dead p110α allele in part resemble *Irs1* null mice displaying growth retardation, insulin resistance, but with improved glucose homeostasis at older ages [43,44]. These studies suggest a dominant role for p110α in mediating the physiological actions of *Irs1* rather than *Irs2*. However, epistasis studies crossing mice heterozygous for *Foxo1* (a key PI3K- and AKT-regulated transcription factor) with global *Irs2* null mice, partially rescued the defective β-cell phenotypes seen in this animal model [45]. Taken together, such studies have therefore provided incomplete insights into the role of specific signals downstream of IRS1 and IRS2 in mediating physiological effects.

To directly address the role of specific signals recruited by IRS1 and IRS2, we generated a series of knock-in mice lacking the ability to recruit either PI3K or GRB2 to IRS1 or IRS2. We undertook detailed signalling and metabolic phenotyping of these animals. These studies revealed that PI3K recruitment to IRS1 or IRS2 is the major signalling pathway mediating the physiological effects of these signalling molecules. In contrast, GRB2-mediated signalling plays a minimal role in growth and metabolism.

## 2 Methods and materials

### 2.1 Construction and expression of mutated IRS proteins

A mouse *Irs2* cDNA in pBluescript (Agilent Technologies) was modified by PCR amplification to include a c-Myc tag immediately prior to the stop codon, followed by a novel *NotI* restriction site. Mutated *Irs2* cDNAs were generated using the QuikChange Multi-Site-Directed Mutagenesis kit (Agilent Technologies) according to the manufacturer's instructions. After creating up to three mutations in a single round of mutagenesis, the subsequent cDNA was used in further rounds of mutagenesis until it contained mutations that replaced all of the tyrosine residues of interest with phenylalanine (Tyr-536, 538, 594, 649, 671, 734, 758, 814, 911, 970, 1061, 1242, 1303). The presence of desired mutations was confirmed by DNA sequencing (TAT (Y) to TTT (F) and TAC (Y) to TTC (F)). Mutant cDNAs were then subcloned into the *BamHI*/*NotI* sites of the expression vector pIRES2-EGFP (Clontech). At this stage, the complete sequence of the *Irs2* cDNA was determined to confirm mutagenesis and exclude any mutations introduced by replication error. *Irs2* expression constructs (c-Myc-tagged *Irs2*) were then transfected into Chinese hamster ovary cells stably expressing the insulin receptor (CHO-IR, a gift from TS Pillay, maintained as previously described [46]) using GeneJuice (Novagen). CHO-IR cells were serum-starved overnight and subsequently treated with 100 nM insulin for 5 min for functional *in vitro* signalling studies.

### 2.2 Immunoprecipitation of IRS1 and IRS2 proteins

Antibodies detecting IRS1 or IRS2 were generated by creating Glutathione-S-transferase (GST) fusion proteins containing either residues 735–900 of murine IRS1 or residues 619–746 of murine IRS2 subcloned into the pGEX6p1 GST-expression vector system (GE Healthcare). Expression was induced by the addition of 1 mM IPTG at 37 ^o^C for 2 h in BL-21 cells (Thermo Fisher Scientific) with GST-IRS1 and GST-IRS2 fusion proteins purified using Redipack GST columns (GE Healthcare) via elution using 10 mM glutathione. Fusion proteins were desalted using HiTrap columns (GE Healthcare) following manufacturer recommended protocol prior to injections into sheep at the Division of Signal Transduction Therapy (DSTT), College of Life Sciences, University of Dundee to generate anti-IRS1 and anti-IRS2 antisera.

CHO-IR cells were homogenised in lysis buffer (50 mM Tris–HCl pH 7.5, 150 mM NaCl, 1% Triton-X100, 1 mM EDTA, with Complete Mini protease inhibitors and PHOSstop phosphatase inhibitors (both Roche)). Cleared lysates were assessed for total protein content by the BCA method (Bio-Rad) and 1 mg of total protein immunoprecipitated with 10 μL sheep polyclonal anti-IRS1 or anti-IRS2 antibodies and protein G-Sepharose beads (Sigma–Aldrich) (total volume 1 mL), rotating overnight at 4 °C. Beads were washed four times with lysis buffer, boiled in Laemmli buffer and analysed by Western blotting as below.

### 2.3 PI3K assay

For assessment of PI3K activity, washed beads from immunoprecipitates (as above) were further washed with a 2x kinase assay buffer (40 mM Tris–HCl pH 7.5, 200 mM NaCl, 1 mM EGTA) and resuspended in 60 μL of 1x kinase assay buffer plus 10 mM MgCl_2_, 100 μM ATP (containing 0.5 μCi of [γ^32^P] ATP per sample) and 200 μg/mL phosphatidylinositol as a substrate. Reactions were incubated for 20 min at 25 °C and terminated by the addition of 100 μL of 0.1 M HCl. 200 μL 1:1 chloroform/methanol was added to each sample, vortexed and centrifuged at 12,000×*g* for 2 min. The organic phase was washed with 80 μL 1:1 1M HCl/methanol, recentrifuged, and the lipids from the organic phase were dried by evaporation. Dried lipids were resuspended in 30 μL 4:1 chloroform/methanol and spotted onto thin-layer Silica Gel-60 plates (Merck) that had been pretreated with 1% oxalic acid, 1 mM EDTA in 40% methanol. Plates were separated in 13:7 n-propanol/2 M acetate, dried, exposed to autoradiography, and captured using a FLA-2000 phosphoimager (Fuji), with band intensities quantified by densitometry (ImageJ), all as previously described [47].

### 2.4 Animals and diets

ARRIVE guidelines were used for the designing and reporting of animal experiments [48]. Where possible, investigators were blinded to the genotype of both live animals and tissue/blood samples. For feeding studies, mice were randomised by genotype within study groups, and a crossover design was used. All metabolic studies were replicated across at least two (typically three or more) independent cohorts. Key physiological parameters were measured in both male and female mice, with tissues from male mice used for downstream assessment of protein expression and binding assays.

Mice with knock-in alleles, with conversion of tyrosine residues with potential p85 binding sites or potential GRB2 binding sites to phenylalanine residues inserted into the endogenous locus of either *Irs1* or *Irs2*, were generated by homologous recombination in embryonic stem (ES) cells (Taconic Biosciences) using standard approaches. For IRS1, mutant residues were chosen based on the published literature, and for IRS2, the choices were based on our *in vitro* studies combined with the IRS1 literature, by mapping equivalent residues in both molecules. In brief, targeting vectors containing the relevant regions with the mutated *Irs1* and *Irs2* genes were constructed, containing a neomycin cassette flanked by FRT sites as a selectable marker. DNA from targeted C57BL/6NTac ES cells were screened by Southern blotting and PCR. ES cells were subsequently microinjected into BALB/c blastocysts, which were implanted into pseudopregnant females, and chimeric male mice were screened for germline transmission. FLP-mediated excision to delete the neomycin selection cassette was performed *in vivo* by breeding mutant animals with ubiquitous *Flp* recombinase-expressing deleter mice, to produce *Irs1* or *Irs2* knock-in mice for p85 (*Irs1*^+/YnF^ or *Irs2*^+/YnF^) and GRB2 (*Irs1*^+/Y891F^ or *Irs2*^+/Y911F^) (Taconic Biosciences). All mouse lines were backcrossed and maintained in our transgenic animal facility on either a mixed 129S2/Sv:C57BL/6J (*Irs1*^+/YnF^) or a pure C57BL/6J (*Irs2*^+/YnF^, *Irs1*^+/Y891F^, and *Irs2*^+/Y911F^) genetic background. The presence of the mutant allele was determined by PCR analysis of ear tissue biopsy samples. Primers used for amplification were: *Irs1*: 5′-GGGACTCCTGCAATACTT-3′ and 5′-GTGTGTGTTCTCTACATAATCAGG-3′, *Irs2*: 5′-ATGCCACTCTTACTAGTGCTTCCTTGG-3′ and 5′-ACAACTTTCCGCTCTGTAGCACCTAC-3’.

Experimental cohorts of group-housed, young adult (8–12 weeks old) male (unless otherwise stated) mice and their wild type littermates were maintained on a 12-hour light/dark cycle with free access to water and standard mouse chow (RM3, Special Diet Services) and housed in specific pathogen-free barrier facilities. All animal work was carried out in accordance with the UK Animals (Scientific Procedures) Act (1986) as well as being approved by the Imperial College Animal Welfare and Ethical Review Body and by the UK Home Office.

### 2.5 Metabolic analysis

Body weight was measured, tail vein blood was collected, and blood glucose levels were determined using a Contour glucometer (Bayer) as previously described [49]. Intraperitoneal glucose (2 g/kg, overnight fast) and insulin (0.75 IU/kg, overnight fast ± 6 h refeed, where described) tolerance tests, as well as intraperitoneal glucose-stimulated insulin secretion (GSIS) (3 g/kg) were performed as described in [49], with blood collection at the times indicated in the figures. Assessment of food intake was performed in singly housed mice for three consecutive experimental days as previously described [50]. Levels of serum mature insulin, leptin and adiponectin were determined by ELISA (Crystal Chem, Millipore and Crystal Chem, respectively). Triglycerides were quantified in liver and serum using a commercially available assay kit (Cayman Chemical). Fat mass was quantified using an EchoMRI Quantitative Whole-Body Composition analyser (Zinsser Analytic) on unanaesthetised animals.

### 2.6 Hyperinsulinaemic-euglycaemic clamp studies

Clamps were performed as previously described [51,52]. Female mice were fasted overnight and anaesthetised by intraperitoneal injection with 6.25 mg/kg acetylpromazine, 6.25 mg/kg midazolam and 0.31 mg/kg fentanyl. The tail vein was cannulated with 27G needles, and D-[^3^H]-glucose was infused at a rate of 0.36 MBq/hour for 1 h to achieve steady-state levels. Insulin (Novo Nordisk) was infused at a constant rate of 0.09 mU/min after a bolus dose of 3.3 mU, and D-[^3^H]-glucose infusion continued at a rate of 0.36 MBq/hour. A variable infusion of 12.5% glucose was supplied to maintain euglycaemia. 50 mins after initial insulin infusion, ^14^C-2-deoxyglucose (specific activity: 9.25–13.0 GBq/mmol) was administered intravenously to assess specific glucose uptake. Mice were culled by cervical dislocation, and organs were removed and frozen for further analysis. To measure [^3^H]-glucose in serum, proteins were removed by precipitation with trichloroacetic acid (final concentration 2%) and centrifugation, the supernatant was dried and resuspended in ddH_2_O. Radioactivity was measured in these samples using scintillation counting (Hidex Scintillation, LabLogic).

Tissues were later homogenised in 0.5% perchloric acid, followed by centrifugation and neutralisation of the supernatants; ^14^C-2-deoxyglucose was precipitated using BaOH/ZnSO_4_. Total and precipitated counts from supernatants, and plasma, and ^14^C-2-deoxyglucose counts, were used to determine tissue-specific uptake. Protein content in homogenates was determined using the DC protein assay (Bio-Rad) and served as a normalisation factor. Glucose turnover rate (μmol/min) was calculated during the basal period and under steady-state clamp conditions as the rate of tracer infusion (dpm/min) divided by the plasma specific activity of [^3^H]-glucose (dpm/μmol). Hyperinsulinaemic hepatic glucose production was calculated as the difference between the tracer-derived rate of glucose appearance and the glucose infusion rate.

### 2.7 Histological techniques and immunofluorescence

Dissected pancreata were fixed in Bouin's solution, embedded in paraffin and cut into 5 μm sections. Insulin (mouse monoclonal, I2018, clone K36AC10, Sigma–Aldrich) and glucagon (rabbit polyclonal, ab92517, clone EP3070, Abcam) immunostaining, and morphometric analysis were performed blindly as previously described [49] using a DMRB Fluorescence microscope (Leica).

### 2.8 Primary islet isolation and GSIS

For islet isolation, the pancreas was injected with Liberase TM (Roche), digested at 37 °C, purified using a Histopaque gradient (Sigma–Aldrich) and handpicked as previously described [49]. For secretion studies, isolated islets were cultured overnight in RPMI supplemented with 10% FBS. Following a 30-minute pre-incubation in Krebs-HEPES-Bicarbonate buffer (KHB, 140 mM NaCl, 3.6 mM KCl, 0.5 mM NaH_2_PO_4_, 0.2 mM MgSO_4_, 1.5 mM CaCl_2_, 10 mM HEPES, 25 mM NaHCO_3_) with 3 mM glucose, GSIS was assessed by static incubation of six islets (in triplicate, per mouse prep) in KHB with 3 mM or 16.7 mM glucose for 30 min at 37 °C. Secreted mature insulin into the media, and the total mature insulin content of the islets was assayed by ELISA (Crystal Chem).

### 2.9 MEF primary cultures

Primary cultures of mouse embryonic fibroblasts (MEFs) from E12.5 embryos were grown and expanded in 1:1 DMEM/F12, 10% FBS, 2 mM l-Glutamine, 37 °C, 5% CO_2_. Overnight serum-starved MEFs were treated with insulin (1 μM, Novo Nordisk) for 5 min prior to protein extraction and immunoprecipitation as above.

### 2.10 Hepatocyte isolation and culture

Mice were anaesthetised with ketamine (200 mg/kg body weight) and the hepatic portal vein cannulated using a 23G needle, with a 15 min perfusion (5 mL/min, Ismatec, ISM931C) with prewarmed (55 °C) perfusion buffer (10 mM NaHCO_3_, 100 mM HEPES, 0.5 mM EGTA, pH 7.4 in HBSS (Sigma–Aldrich)) followed by perfusion with collagenase buffer (0.05 mg/mL collagenase H (Roche), 1 mM CaCl_2_ in HBSS). The liver was then collected, partially minced in warm HBSS, and the released hepatocytes were passed through a 100 μm cell strainer into warm HBSS. Hepatocytes were twice centrifuged for 5 min at 100×*g* and resuspended in HBSS, and finally resuspended in cold M199 media (Gibco) before being plated in an adherence media (M199 media with 0.1% BSA, 2% UltroSer G (Pall BioSepra), 100 nM T3 (Sigma–Aldrich), 100 nM dexamethasone (Sigma–Aldrich) and 100 nM insulin (Sigma–Aldrich)). 4 h later, after cells had adhered, the media was replaced with standard M199 growth media and incubated overnight. The following day, hepatocytes were treated with insulin (100 nM) for the times indicated, all as previously described [53].

### 2.11 Western blotting analysis

Cells and snap-frozen tissues were homogenised directly into protein lysis buffer (50 mM Tris–HCl, pH 7.5, 150 mM NaCl, 1% Triton X-100, 1 mM EDTA with protease and PHOSstop phosphatase inhibitors (both Roche)). Protein expression was analysed by Western blotting of clarified lysates normalised for protein content by the BCA method (Bio-Rad) as previously described [49], using the primary antibodies listed in Supplemental Table 1.

### 2.12 Statistical analyses

Data sets were analysed for normal distribution using the D'Agostino-Pearson normality test. Statistical significance between groups was determined using GraphPad Prism 7 by 2-tailed Student's *t* test, Mann–Whitney *U* test or ANOVA. The Mann–Whitney *U* test or the Kruskal–Wallis test was used as a nonparametric alternative. Bonferroni (or Dunn's for nonparametric) and Geisser-Greenhouse post hoc tests to correct for multiple comparisons and repeated measures were performed where required. A probability of error less than 5% was considered significant (i.e., *p* < 0.05), and general statistical information for individual experiments (*p*-values and *n*-values) can be found in the figure legends.

## 3 Results

### 3.1 Generation of knock-in mice with IRS1 or IRS2 rendered unable to recruit PI3K

Previous biochemical and bioinformatic studies have identified 9 tyrosine residues in potential p85 binding sites (six with YMXM motifs at positions 608, 628, 658, 727, 935 and 983 and 3 with YXXM motifs at positions 460, 546 and 1006) within the cytoplasmic tail of mouse IRS1 [16,38,54]. Functional studies have partially characterised p85 binding *in vitro*: deletion of all tyrosine residues in IRS1 results in loss of PI3K recruitment, whereas an IRS1 retaining three YMXM motifs at Y608, Y628 and Y658 fully activates PI3K in 32D cells [38]. In mouse IRS2, there are 10 tyrosine residues in potential p85 binding sites (four with YMXM motifs at positions 538, 649, 671 and 734, five with YXXM motifs 536, 594, 758, 814 and 1061 and a YXXL motif at position 970, which is homologous to residue 935 in IRS1). Y649, Y671, Y734 and Y814 have been previously identified as being essential for the association of the PI3K regulatory subunit p85 with IRS2 [55]. However, no systematic functional studies have been performed on IRS2-mediated PI3K recruitment. Therefore, we initially generated a series of c-Myc-tagged IRS2 mutants designed to abrogate this event and expressed them in CHO-IR cells. Specifically, we generated a mutant which we termed p99 which carried the mutations Y536F, Y538F, Y594F, Y649F, Y671F, Y734F, Y758F, Y814F, Y970F and Y1061F and would be predicted to fully ablate PI3K-binding. We also studied two intermediate mutants termed p79 (carrying Y536F, Y538F, Y594F, Y649F, Y671F, Y758F, Y814F, Y970F and Y1061F) and p97 (carrying Y594F, Y649F, Y671F, Y814F and Y970F). In insulin-stimulated CHO-IR cells, IRS2-associated PI3K activity in mutants p79 and p97 was 12% and 41% of the activity observed in cells expressing wild type IRS2 (Supplementary Fig. 1). However, in the p99 mutant, insulin-stimulated IRS2-associated PI3K activity was essentially undetectable (Supplementary Fig. 1).

Based on these *in vitro* biochemical studies, the existing literature, and comparisons of *Irs1* and *Irs2* sequences and motifs, we next generated a pair of constitutive knock-in mice using standard gene targeting approaches, in which all relevant residues involved in PI3K binding were mutated. *Irs1* knock-in mice were designated *Irs1*^YnF/YnF^, carrying the following mutations Y460F, Y546F, Y608F, Y628F, Y658F, Y727F, Y935F, Y983F and Y1006F and *Irs2* knock-in mice designated *Irs2*^YnF/YnF^ carrying the mutations Y536F, Y538F, Y594F, Y649F, Y671F, Y734F, Y758F, Y814F, Y970F and Y1061F (Supplementary Fig. 2A and B).

Previous studies of IRS1 have identified a single GRB2-binding site at tyrosine Y891 [23], and the equivalent residue in IRS2 is Y911 [17,56]. We therefore also generated a pair of constitutive knock-in mice using similar gene-targeting approaches, in which the IRS1 allele carried the Y891F mutation and the IRS2 allele carried the Y911F mutation (Supplementary Fig. 3A and B).

Signalling events were studied in these mice, which were then subjected to a broad range of phenotyping approaches to assess the roles of IRS1- or IRS2-dependent recruitment of PI3K activity and IRS1- or IRS2-dependent GRB2 signalling in whole-animal physiology.

### 3.2 *Irs1*^YnF^ mice are unable to recruit PI3K and display limited or no compensatory alteration in IRS2 expression or signalling

Mice heterozygous for the *Irs1*^YnF^ allele were viable and were intercrossed to produce homozygote embryos that were used to derive wild type and homozygote (herein *Irs1*^YnF^) mouse embryonic fibroblasts (MEFs) for signalling studies. In *Irs1*^YnF^ MEFs, IRS1 and IRS2 expression was equivalent to that seen in control cells, indicating no compensatory alteration in IRS2 levels in contrast to that seen in global *Irs1* null mice [57,58] (Supplemental Fig. 4A). Expression of p110α and p85 subunits of PI3K was also unaltered (Supplemental Fig. 4A). Next, we assessed the signalling response to insulin. As anticipated, there was no p85 binding in *Irs1*^YnF^ MEFs, but GRB2 and SHC recruitment to IRS1 was preserved (Figure 1A). Consistent with these findings, insulin treatment did not increase PI3K activity in IRS1 immune complexes from *Irs1*^YnF^ MEFs compared to control cells (Figure 1B). In contrast, insulin-stimulated IRS2-associated PI3K activity in *Irs1*^YnF^ MEFs was equivalent to that seen in control cells, indicating normal levels of IRS2-signalling with no compensatory increase in PI3K activity (Figure 1B). We further explored insulin signalling in tissues derived from homozygote *Irs1*^YnF^ mice. In *Irs1*^YnF^ MEFs, activating phosphorylation of AKT by insulin (both S473 and T308) was significantly reduced (Supplemental Fig. 4D–F). Expression of IRS1 in liver and brain was equivalent in *Irs1*^YnF^ and control mice but there was a slight increase in IRS2 expression (Supplemental Fig. 4B and C). Increased expression of IRS1 and IRS2 was also observed in hepatocytes isolated from *Irs1*^YnF^ mice (IRS1: FC = 1.58, *p* = 0.0070; IRS2: FC = 1.76, *p* = 0.0096, *n* = 5). Activating phosphorylation of AKT by insulin was preserved in hepatocytes from this mutant (Figure 1C, Supplemental Fig. 4G). Likewise, phosphorylation of p42/44 ERK in response to insulin was also unchanged (Figure 1C, Supplemental Fig. 4H). Taken together, these signalling studies indicate specific loss of IRS1-dependent PI3K activation in *Irs1*^YnF^ mice in tissues with limited compensation or changes in expression of IRS 1 or IRS2 in a cell specific manner, and no alteration in IRS2-associated PI3K activity in MEFs, thus making them a suitable model to explore the contribution of this specific signalling pathway in whole animal physiology.

**Figure 1:**
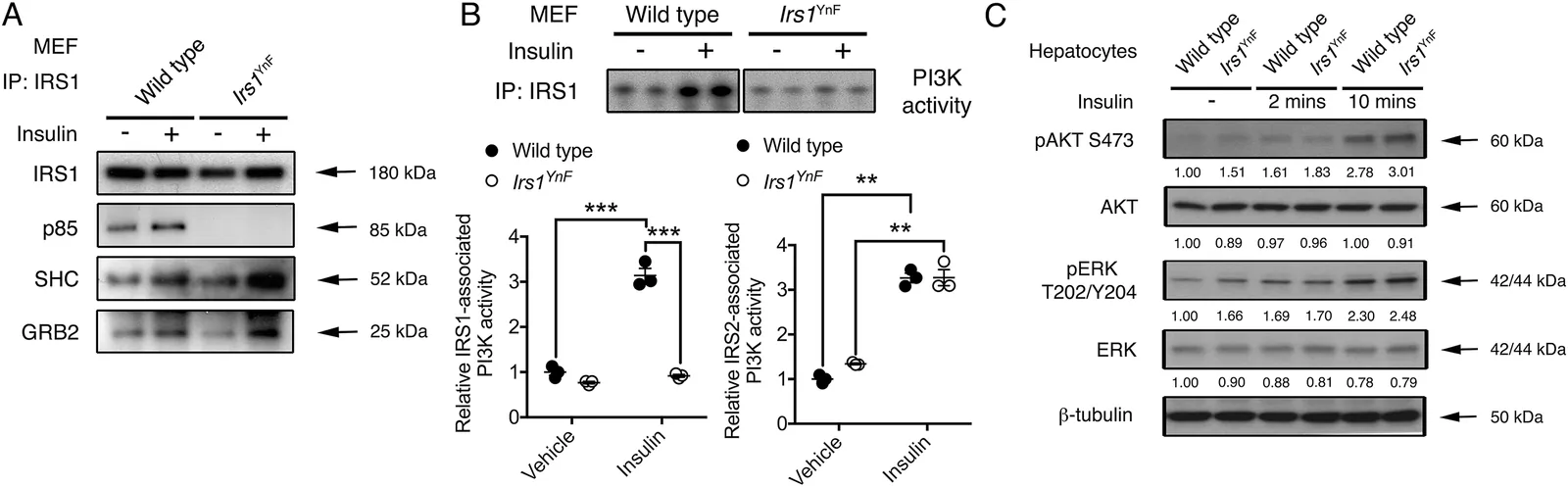
**IRS1 does not bind PI3K in primary cells from *Irs1*^YnF^ mice.** (A) Lysates from *Irs1*^YnF^ insulin-treated MEFs were immunoprecipitated (IP) using anti-IRS1 antibodies. Proteins in IPs were detected by Western blotting using antibodies against IRS1, p85, SHC and GRB2. The panel shows a representative blot from three independent experiments. (B) IPs as in A were assayed for PI3K activity using radiolabelling with [γ^32^P] ATP and subsequent autoradiography. The panel shows a representative autoradiograph of three independent experiments. Mean band intensities from multiple experiments quantified by densitometry are shown in the associated bar charts, expressed relative to wild type/vehicle samples (*n* = 3). (C) Representative Western blotting analysis of protein levels in insulin-treated primary hepatocytes from *Irs1*^YnF^ and wild type mice (*n* = 4 mice per genotype). β-Tubulin was used as a loading control. Mean band intensities from multiple experiments quantified by densitometry are shown below each panel, all expressed relative to wild type/untreated samples. Data is represented as mean ± SEM (⁎⁎*p* < 0.01, ⁎⁎⁎*p* < 0.001).

### 3.3 *Irs1*^YnF^ mice are growth restricted and display hepatic insulin resistance, particularly in the refed state

On a pure C57BL/6J background, heterozygote *Irs1*^YnF^ intercrosses produced homozygote *Irs1*^YnF^ mice with poor viability at ratios below the expected Mendelian distribution, consistent with previous phenotypic observations in global *Irs1* null mice (data not shown). We therefore studied these animals on a mixed C57BL/6J:129S2/Sv background on which mice of each genotype were born with expected frequencies (wild type 26%, heterozygous 51%, homozygous 23%). *Irs1*^YnF^ mice of both sexes displayed a 40% reduction in weight at birth and this growth retardation persisted into adulthood (Figure 2A,B). Organ weight was also reduced in female *Irs1*^YnF^ mice, but when expressed as a percentage of bodyweight, the brain and other organs were relatively larger (Figure 2C,D). Together, this data suggests that IRS1-dependent PI3K signalling plays a key role in somatic growth including during development, consistent with the role for IRS1 revealed by global gene targeting [[28], [29], [30]].

**Figure 2:**
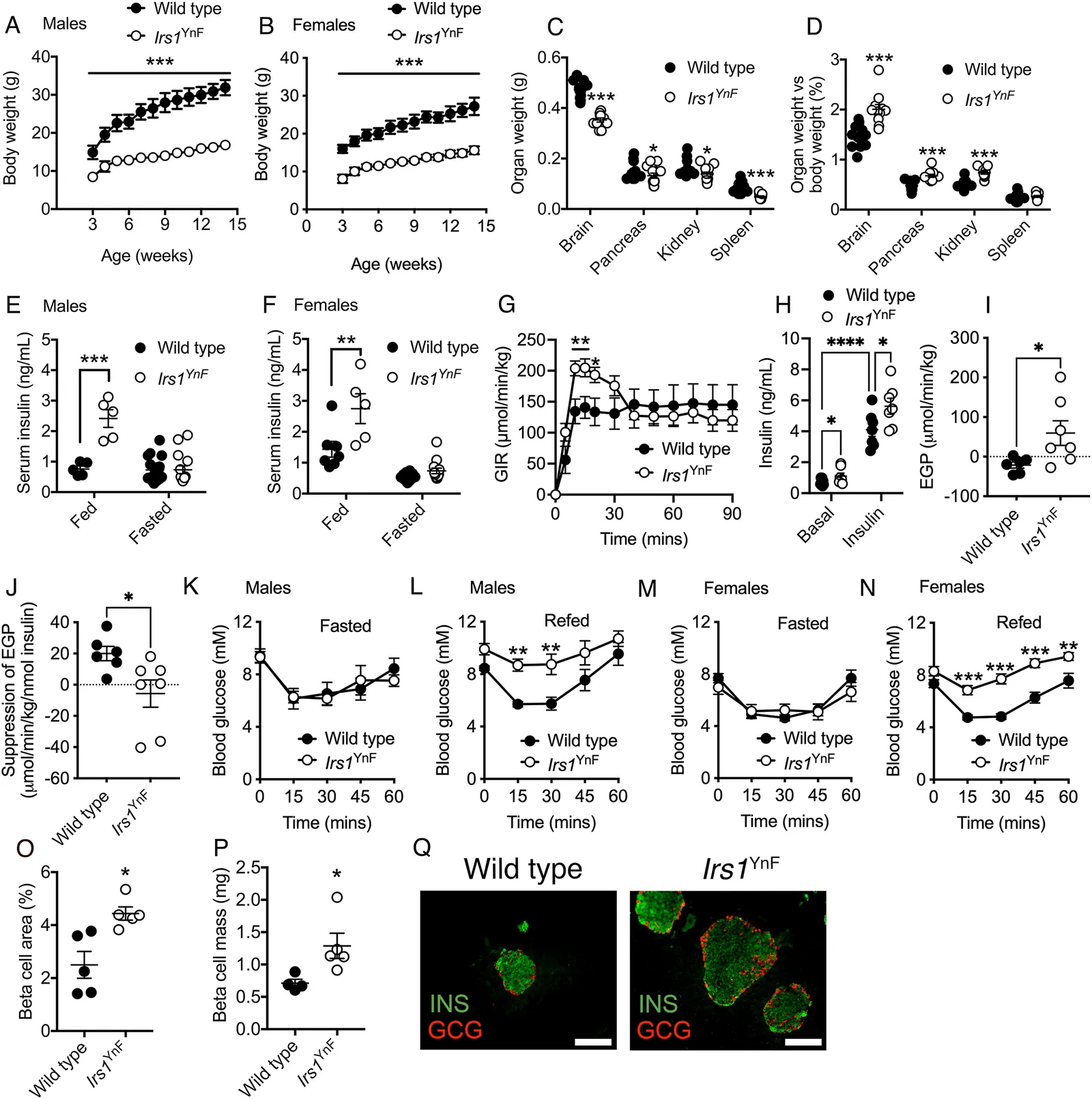
**Growth restriction and hepatic insulin resistance in *Irs1*^YnF^ mice.** (A) Body weights of male (A) and female (B) wild type and *Irs1*^YnF^ mice from weaning age (3 weeks) to 14 weeks (*n* = 6 mice). (C, D) Raw organ weights (C) and organ to body weight ratios (D) of adult female wild type and *Irs1*^YnF^ mice (*n* = 13 mice per genotype). (E, F) Serum insulin levels in adult male (E) and female (F) wild type and *Irs1*^YnF^ mice in both the fed and fasted state (*n* = 5–7 for fed and *n* = 15–18 for fasted, per genotype). (G) Glucose infusion rate (GIR) in adult female wild type and *Irs1*^YnF^ mice (*n* = 7 and 9 for wild type and *Irs1*^YnF^ mice respectively). (H) Serum insulin levels at basal and during hyperinsulinaemic-euglycaemic (insulin) conditions (n = 7 per genotype). (I, J) Endogenous glucose production (EGP) in adult female wild type and *Irs1*^YnF^ mice normalised to bodyweight (I) and bodyweight/serum insulin (J) (*n* = 6 and 7 for wild type and *Irs1*^YnF^ mice respectively). Suppression of EGP in J indicates the hepatic insulin sensitivity index calculated as: (basal EGP - clamp EGP)/clamp insulin concentration. (K–N) Insulin tolerance testing in male and female adult wild type and *Irs1*^YnF^ mice in both the fasted and refed state (*n* = 5 per genotype). (O–Q) Morphometric analysis of β-cell area (O) and β-cell mass (P) in pancreatic sections from adult male wild type and *Irs1*^YnF^ mice (*n* = 5 per genotype) with representative immunofluorescent images (Q) showing staining of insulin (INS, green) and glucagon (GCG, red). Scale bar = 50 μm. In all panels, data are represented as mean ± SEM (⁎*p* < 0.05, ⁎⁎*p* < 0.01, ⁎⁎⁎*p* < 0.001, ⁎⁎⁎⁎*p* < 0.0001).

*Irs1*^YnF^ mice of both sexes displayed normal fasting and fed blood glucose levels (Supplemental Fig. 5A and B). Furthermore, glucose tolerance testing was normal in both male and female animals (Supplemental Fig. 5C and D). These findings are consistent with observations in global *Irs1* null mice which display minimal alterations in glucose handling in these tests despite insulin resistance, due to compensatory hyperinsulinemia [28,29]. In addition, we observed elevated insulin levels in the fed state in male and female *Irs1*^YnF^ mice, but surprisingly, in the fasted state, insulin levels were normal (Figure 2E,F). We performed hyperinsulinemic-euglycemic clamps to assess insulin resistance in *Irs1*^YnF^ mice. Steady-state conditions were achieved during the final 30 min (t = 60–90 min) (Figure 2G and Supplemental Fig. 5E). When expressed as body weight-normalized glucose infusion rate, accounting for differences in body mass between genotypes, *Irs1*^YnF^ mice displayed comparable overall insulin sensitivity to wild type controls during the steady-state period (Figure 2G). Tracer studies revealed that the basal rate of glucose disposal (equivalent to basal glucose production) and glucose disposal under hyperinsulinemic conditions were equivalent in control and *Irs1*^YnF^ mice (Supplemental Fig. 5F). However, in the hyperinsulinemic state, suppression of endogenous glucose production was significantly impaired, a deficit that was preserved when adjusting for serum insulin (Figure 2H–J). In *Irs1*^YnF^ mice, 2-deoxyglucose uptake into muscle and white adipose tissue under hyperinsulinemic conditions was comparable to that of control mice, suggesting a lack of any marked insulin resistance phenotype in these tissues (Supplemental Fig. 5G). Together, these data suggest that *Irs1*^YnF^ mice display marked hepatic insulin resistance rather than defects in skeletal muscle and adipose tissue, which contrasts with our earlier clamp findings in global *Irs1* null mice, which predominantly showed skeletal muscle insulin resistance and mild liver defects [32]. However, subsequent studies in mice with liver-specific deletion of *Irs1* have shown differential roles for *Irs1* in liver metabolism, with these animals manifesting insulin resistance and increased hepatic glucose production in the refed state but not in the fasted state [59]. To further investigate this, we performed insulin tolerance tests in fasted and refed *Irs1*^YnF^ mice. These studies showed that fasted *Irs1*^YnF^ mice cleared glucose normally while after refeeding, *Irs1*^YnF^ mice displayed impaired glucose disposal, indicating insulin resistance (Figure 2K-N).

In *Irs1* global null mice, there is a marked compensatory response in pancreatic β-cell mass in the face of systemic insulin resistance [31,60]. *Irs1*^YnF^ mice likewise displayed increased β-cell area and β-cell mass (Figure 2O-Q). However, in contrast to *Irs1* null islets [61] studied *in vitro*, islets from *Irs1*^YnF^ mice did not show an alteration in glucose-stimulated insulin secretion (Supplemental Fig. 5H)**.**

### 3.4 *Irs1*^YnF^ mice are lean and display relatively increased food intake but have normal fertility

*Irs1* global null mice have reduced adiposity and altered feeding behaviour [33]. Consistent with these observations, *Irs1*^YnF^ mice displayed reduced adiposity, reduced lean mass and a reduced fat mass/lean mass ratio (Supplemental Fig. 5I–K). These animals were also hypoleptinaemic (Supplemental Fig. 5L), although this was proportional to the observed reduction in fat mass (Supplemental Fig. 5M, *p* = 0.82). *Irs1*^YnF^ mice showed increased food intake when measured relative to either bodyweight or lean mass, thereby resembling *Irs1* global null mice (Supplemental Fig. 5N-P). Levels of triglycerides in the liver were unaltered in both male and female *Irs1*^YnF^ mice, with serum triglycerides significantly reduced in male *Irs1*^YnF^ mice (Supplemental Fig. 5Q–S). Adiponectin levels in serum were increased in male *Irs1*^YnF^ mice (Supplemental Fig. 5T and U). Consistent with findings in *Irs1* global null mice [36], *Irs1*^YnF^ mice on a hybrid C57BL/6J:129S2/Sv background displayed normal fertility parameters (data not shown). Taken together, these studies indicate that PI3K signalling specifically recruited by IRS1 appear to mediate the predominant effects on metabolism and growth in mice totally lacking IRS1.

### 3.5 *Irs2*^YnF^ mice are unable to recruit PI3K and display no compensatory alteration in IRS1 expression or signalling

Mice heterozygous for the *Irs2*^YnF^ allele were viable and were intercrossed to produce homozygote embryos that were used to derive wild type and *Irs2*^YnF^ MEFs for signalling studies. IRS1 and IRS2 expression was equivalent to that seen in control cells indicating no compensatory alteration in IRS1 levels (Supplemental Fig. 6A). Expression of p110α and p85 was also unaltered (Supplemental Fig. 6A). In response to insulin stimulation, no p85 was recruited to IRS2 in *Irs2*^YnF^ MEFs, while GRB2 and SHC recruitment was preserved (Figure 3A). Consistent with these findings, insulin treatment did not increase PI3K activity in IRS2 immune complexes from *Irs2*^YnF^ MEFs compared to control cells (Figure 3B). Furthermore, IRS1-associated PI3K activity was preserved indicating normal IRS1 signalling without evidence of compensation (Figure 3B). In *Irs2*^YnF^ MEFs, activating phosphorylation of AKT by insulin (both S473 and T308) was significantly reduced (Supplemental Fig. 6D–F). Expression of IRS1 and IRS2 in liver and brain was equivalent in *Irs2*^YnF^ and control mice (Supplemental Fig. 6B and C). In isolated *Irs2*^YnF^ hepatocytes, activation of AKT by insulin was attenuated while p42/44 ERK activation was preserved (Figure 3C, Supplemental Fig. 6G and H). In contrast to hepatocytes isolated from *Irs1*^YnF^ mice, we did not observe any altered expression of IRS1 or IRS2 in *Irs2*^YnF^ hepatocytes (IRS1: FC = 0.84, *p* = 0.35; IRS2: FC = 1.03, *p* = 0.53, *n* = 6). These studies indicate a specific loss of IRS2-dependent PI3K activation in *Irs2*^YnF^ mice, with no evidence of compensation via IRS1. We subsequently studied the contribution of this specific signalling element to whole animal physiology.

**Figure 3:**
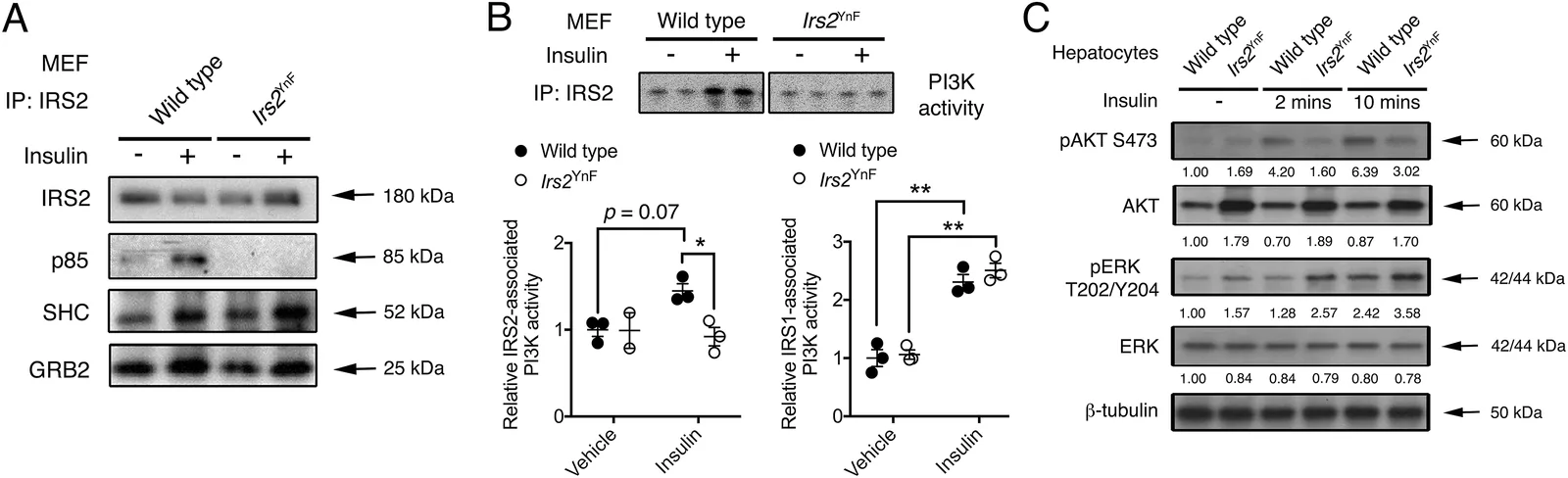
**IRS2 does not bind PI3K in primary cells from*****Irs2***^**YnF**^**mice.** (A) Lysates from *Irs2*^YnF^ insulin-treated MEFs were immunoprecipitated using anti-IRS2 antibodies. Proteins in IPs were detected by Western blotting using antibodies against IRS2, p85, SHC and GRB2. The panel shows a representative blot from three independent experiments. (B) IPs as in A were assayed for PI3K activity using radiolabelling with [γ^32^P] ATP and subsequent autoradiography. The panel shows a representative autoradiograph from three independent experiments. Mean band intensities from multiple experiments quantified by densitometry are shown in associated bar charts, expressed relative to wild type/vehicle samples (*n* = 3). (C) Representative Western blotting analysis of protein levels in insulin-treated primary hepatocytes from *Irs2*^YnF^ and wild type mice (*n* = 4 mice per genotype). β-Tubulin was used as a loading control. Mean band intensities from multiple experiments quantified by densitometry are shown below each panel, all expressed relative to wild type/untreated samples. Data is represented as mean ± SEM (⁎*p* < 0.05, ⁎⁎*p* < 0.01).

### 3.6 *Irs2*^YnF^ mice display sexually dimorphic deteriorations in glucose homeostasis and insulin resistance in the fasted state

On a C57BL/6J background, *Irs2*^YnF/YnF^ (herein *Irs2*^YnF^) mice were born at the expected Mendelian frequencies from heterozygote intercrosses and birth weight was equivalent to littermate controls (data not shown). Postnatal bodyweight profiles were similar in male *Irs2*^YnF^ mice and control animals, whereas female mutant mice were heavier than their controls from 10 weeks of age (Figure 4A,B). Assessment of organ weights showed that brain weight was relatively reduced in *Irs2*^YnF^ mice compared to controls, similar to that seen in *Irs2* global null mice [30] (Figure 4C).

**Figure 4:**
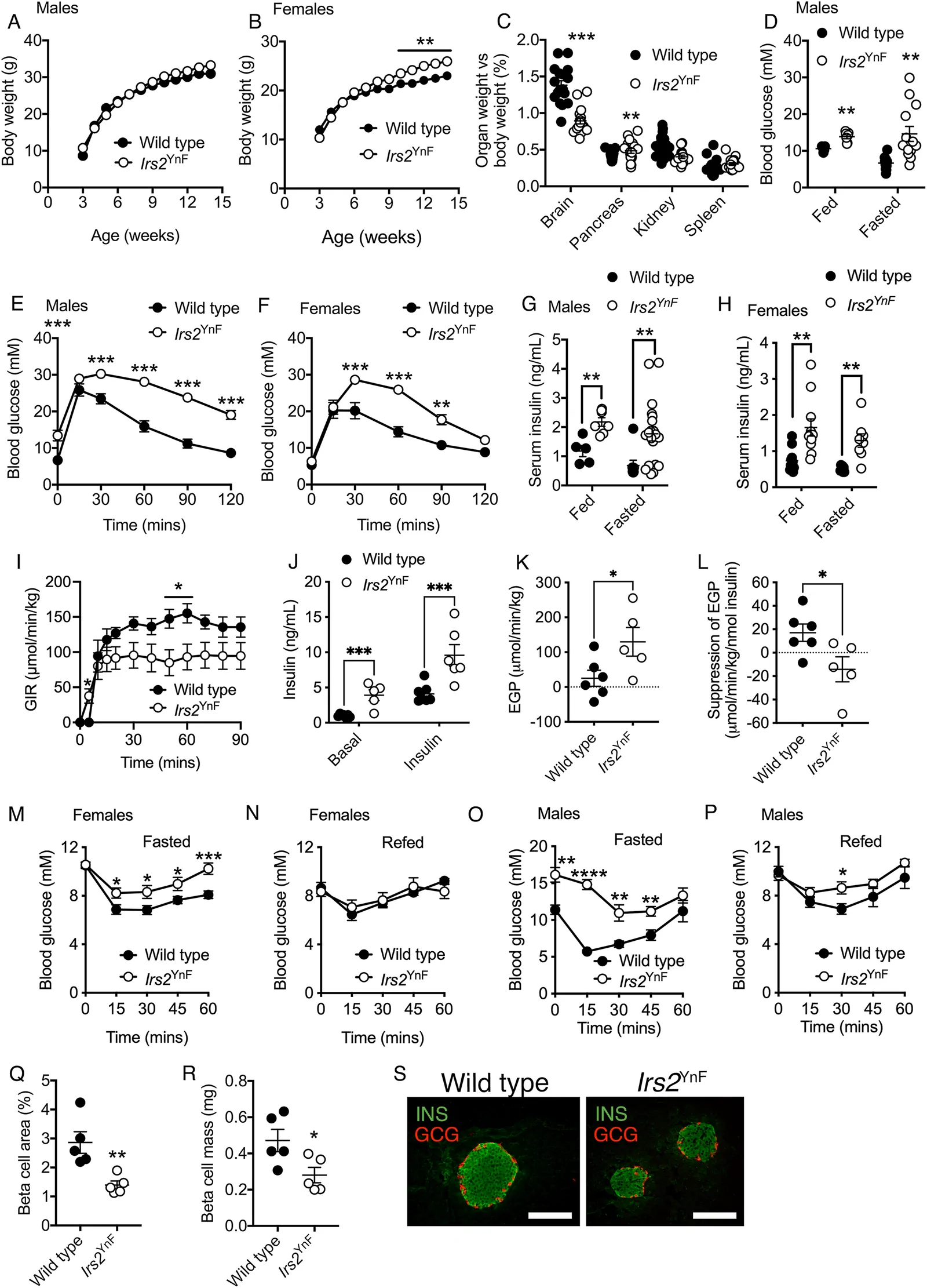
**Sexually dimorphic deterioration in glucose homeostasis in *Irs2*^YnF^ mice.** (A) Body weights of male (A) and female (B) wild type and *Irs2*^YnF^ mice from weaning age (3 weeks) to 14 weeks (*n* = 12 and n = 19 mice for males and females respectively, per genotype). (C) Organ to body weight ratios of adult male wild type and *Irs2*^YnF^ mice (*n* = 17 mice per genotype). (D) Blood glucose levels in adult male wild type and *Irs2*^YnF^ mice in both the fed and fasted state (*n* = 5–7 and *n* = 12–13 mice per genotype for fed and fasted respectively) (E, F) Glucose tolerance testing in adult wild type and *Irs2*^YnF^ mice (*n* = 12 and *n* = 8 per genotype for males and females respectively). (G, H) Serum insulin levels in adult wild type and *Irs1*^YnF^ mice in both the fed and fasted state (males: *n* = 5–7 and *n* = 8–20 per genotype for fed and fasted respectively; females: *n* = 11 and *n* = 7–8 per genotype for fed and fasted, respectively). (I) Glucose infusion rate (GIR) in adult female wild type and *Irs2*^YnF^ mice (*n* = 6 per genotype). (J) Serum insulin levels at basal and during hyperinsulinaemic-euglycaemic (insulin) conditions (n = 6 per genotype). (K, L) Endogenous glucose production (EGP) in adult female wild type and *Irs2*^YnF^ mice normalised to bodyweight (K) and bodyweight/serum insulin (L) (*n* = 7 per genotype). Suppression of EGP in L indicates the hepatic insulin sensitivity index calculated as: (basal EGP - clamp EGP)/clamp insulin concentration. (M–P) Insulin tolerance testing in male and female adult wild type and *Irs2*^YnF^ mice in both the fasted and refed state (*n* = 6–9 per genotype). (Q–S) Morphometric analysis of β-cell area (Q) and β-cell mass (R) in pancreatic sections from adult male wild type and *Irs2*^YnF^ mice (*n* = 5–6 per genotype) with representative immunofluorescent images (S) showing staining of insulin (INS, green) and glucagon (GCG, red). Scale bar = 50 μm. In all panels, data are represented as mean ± SEM (⁎*p* < 0.05, ⁎⁎*p* < 0.01, ⁎⁎⁎*p* < 0.001, ⁎⁎⁎⁎*p* < 0.0001).

Mice with global deletion of *Irs2* demonstrate sexually dimorphic age- and genetic background-dependent abnormalities in glucose homeostasis [31,35,36]. Consistent with this observation, 4 month old male *Irs2*^YnF^ mice displayed elevated fed and fasted blood glucose levels, and marked glucose intolerance (Figure 4D,E). In contrast, female *Irs2*^YnF^ mice had normal fed and fasting blood glucose at 10 weeks of age (Supplemental Fig. 7A), although by 6 months of age, they developed marked glucose intolerance (Figure 4F). Both male and female *Irs2*^YnF^ mice displayed fed- and fasting-induced hyperinsulinaemia, suggesting insulin resistance (Figure 4G,H).

In hyperinsulinemic-euglycemic clamp studies, prior to insulin infusion, *Irs2*^YnF^ mice exhibited marked basal hyperglycemia compared to wild type controls (Supplemental Fig. 7B). During hyperinsulinemic conditions, blood glucose in *Irs2*^YnF^ mice was maintained at a stable, elevated isoglycemic plateau throughout the 90-minute procedure (Supplemental Fig. 7B). Steady-state conditions were successfully established in both groups during the final 30 min (t = 60–90 min), evidenced by stable blood glucose concentrations with minimal intra-individual variance (<5%) (Supplemental Fig. 7B). The persistence of elevated blood glucose in *Irs2*^YnF^ mice despite continuous hyperinsulinemic stimulation highlights the severe impairment of insulin-mediated glucose clearance in this genotype.

Body weight normalized glucose infusion rate was lower in *Irs2*^YnF^ mice, indicative of reduced whole-body insulin sensitivity (Figure 4I). Basal glucose disposal rate and glucose disposal under hyperinsulinemic conditions were equivalent in control and *Irs2*^YnF^ mice (Supplemental Fig. 7C). However, in the hyperinsulinemic state, suppression of endogenous glucose production was significantly impaired, a deficit that was preserved when adjusting for serum insulin (Figure 4J-L). In *Irs2*^YnF^ mice, 2-deoxyglucose uptake into muscle and white adipose tissue under hyperinsulinemic conditions was comparable to that of control mice (Supplemental Fig. 7D). Together, these data suggest that *Irs2*^YnF^ mice display marked hepatic insulin resistance, broadly consistent with our previous observations in global *Irs2* null mice [31,32].

To explore any differential effects of feeding status upon liver metabolism, we performed insulin tolerance tests in both fasted and refed states. These studies showed insulin resistance in fasted but not refed female animals (Figure 4M,N), consistent with data from mice with liver-specific *Irs2* deletion [59]. However, male animals at 4 months of age exhibited abnormal insulin tolerance in both the fasted and fed states, suggesting marked insulin resistance and/or β-cell dysfunction under these conditions (Figure 4O,P).

In *Irs2* global null mice, the development of progressive glucose intolerance and diabetes is, in part, due to the lack of a pancreatic β-cell mass compensatory response to insulin resistance [31,32,35]. Consistent with the abnormal glucose homeostasis seen in *Irs2*^YnF^ mice, these animals also displayed decreased β-cell area and β-cell mass (Figure 4Q–S). GSIS was unaffected in *Irs2*^YnF^ mice, although basal insulin secretion was modestly reduced (Supplemental Fig. 7E).

### 3.7 *Irs2*^YnF^ mice are obese and have reduced fertility

Female *Irs2* global null mice develop obesity and have relatively increased food intake [36]. The development of marked hyperglycemia confounded such analysis in male mice of this genotype. Consistent with the increase in bodyweight seen in female *Irs2*^YnF^ mice, these animals also displayed increased adiposity, unaltered lean mass and an increased fat/lean body mass ratio (Figure 5A, Supplemental Fig. 7F and G). Female *Irs2*^YnF^ mice also had elevated leptin levels (Figure 5B) although this was statistically proportionate to the observed increase in fat mass (Supplemental Fig. 7H, *p* = 0.22). Food intake in these animals was slightly lower than that seen in control mice relative to either bodyweight or fat mass, but not to lean mass, broadly consistent with the increased leptin levels and preservation of leptin signalling pathways in mice lacking *Irs2* in the central nervous system [62] (Figure 5C–E). Levels of triglycerides in the liver were unaltered in male and female *Irs2*^YnF^ mice, and serum triglycerides were unaltered in male *Irs2*^YnF^ mice (Supplemental Fig. 7I–K) while adiponectin levels in serum of male mice were significantly increased (Supplemental Fig. 7L and M). Male *Irs2* null mice have normal fertility prior to the onset of diabetes, and male *Irs2*^YnF^ mice also displayed normal reproductive function (data not shown). Female *Irs2* null mice have significantly impaired fertility (depending on genetic background) with defects primarily in the ovary [36,37]. In contrast, female *Irs2*^YnF^ mice were fertile but had an increased interval between pregnancies, suggesting only a mild defect in reproductive function (wild type: 19.8 ± 0.4 days vs. 31.8 ± 3.9 days in *Irs2*^YnF^ mice, *n* = 5 litters per genotype, *p* = 0.0159).

**Figure 5:**
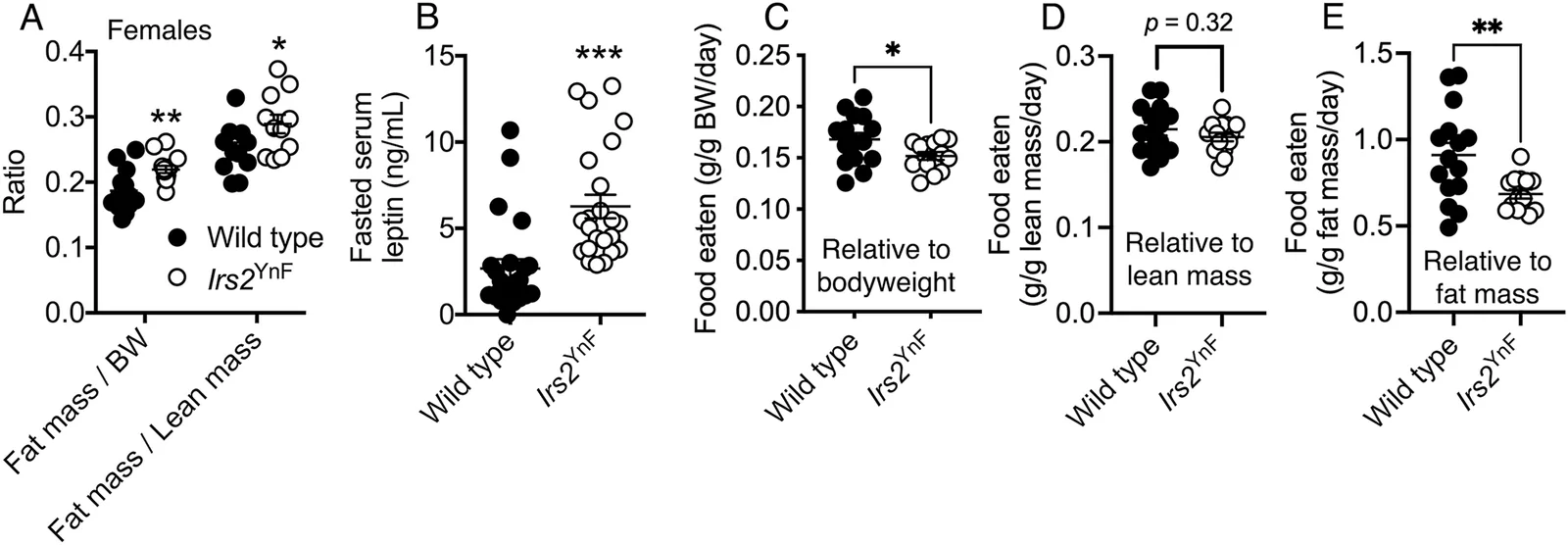
***Irs2*^YnF^ mice have increased adiposity and are hyperleptinaemic.** (A) Adiposity as a ratio of bodyweight (BW) and fat mass vs lean mass in female wild type (*n* = 14) and *Irs2*^YnF^ mice (*n* = 11) quantified by MRI scanning. (B) Fasted serum leptin levels in adult female wild type and *Irs2*^YnF^ mice (*n* = 24 per genotype). (C–E) Food intake in adult female wild type and *Irs2*^YnF^ mice (*n* = 14 per genotype) vs bodyweight (C), lean mass (D) and fat mass (E). In all panels, data are represented as mean ± SEM (⁎*p* < 0.05, ⁎⁎*p* < 0.01, ⁎⁎⁎*p* < 0.001).

### 3.8 Validation and phenotypes of *Irs1*^Y891F^*and Irs2*^Y911F^ mice that are unable to recruit GRB2 to IRS1 and IRS2 respectively

*Irs1*^Y891F^ *and Irs2*^Y911F^ mice were viable and born at expected Mendelian frequencies (data not shown). Glucose tolerance tests, insulin tolerance tests, fasting insulin levels, and body weights were comparable between the two mutants and their respective control mice (Supplemental Fig. 8 A-F and data not shown). To exclude signalling compensation, we therefore intercrossed *Irs1*^Y891F^ *and Irs2*^Y911F^ mice to generate a double mutant *Irs1*^Y891F^/*Irs2*^Y911F^ line. We first undertook a series of signalling studies in MEFs derived from these mice. Expression of IRS1, IRS2 and GRB2 was equivalent between mutant and wild type MEFs (Supplemental Fig. 9A). Insulin-stimulated recruitment of p85 to IRS1 and IRS2 was also preserved, but GRB2 recruitment to IRS proteins was lost (Supplemental Fig. 9B). In isolated hepatocytes, downstream of IRS1 and IRS2, AKT activation was preserved as was ERK activation (Supplemental Fig. 9C). Physiological analysis revealed that *Irs1*^Y891F^/*Irs2*^Y911F^ mice had normal body weight, normal glucose tolerance and insulin sensitivity, and normal fasting insulin levels (Supplemental Fig. 9D–J).

Therefore, abrogation of IRS1 and IRS2-dependent GRB2 signalling had minimal effects on growth and glucose homeostasis in mice. Taken together with data from IRS1^YnF^ and IRS2^YnF^ mutants, these studies indicate that PI3K signalling specifically recruited by IRS1 or IRS2 appears to mediate the predominant metabolic effects observed in mice globally lacking these proteins.

## 4 Discussion

Insulin signalling is a key regulator of organismal metabolism, growth, and survival, mediated by a complex network of intracellular effector molecules. Amongst the earliest and most critical components of this cascade are the IRS proteins, which act as molecular scaffolds linking the activated insulin receptor (IR) to multiple downstream signalling pathways, including the PI3K-AKT and GRB2-MAPK modules. Global deletion of *Irs1* in mice leads to significant growth defects and insulin resistance, yet β-cell compensation results in relatively mild disturbances in glucose homeostasis. *Irs2* null mice are insulin resistant and develop diabetes due to β-cell failure but display a general preservation of somatic growth. However, constitutive deletion models stop the recruitment of all IRS-dependent downstream signalling components and often lead to compensatory adaptations (such as upregulation of other IRS proteins or their adaptor proteins [58]), which may obscure specific mechanistic insights. Similarly, pharmacological inhibition of individual signalling components lacks the specificity to dissect discrete protein–protein interactions within the IRS network. Due to these limitations, the precise physiological roles of each signalling pathway, specifically downstream of IRS1 and IRS2, are not well understood. To overcome these challenges, and maintain IRS and adaptor protein stoichiometry *in vivo*, we generated a series of mutant mice with knock-in alleles of IRS1 and IRS2 that were unable to bind either PI3K or GRB2, thereby bridging the gap between biochemical mechanistic studies and whole-animal physiology. Here, we show that mice lacking IRS1:PI3K binding display significant growth restriction and insulin resistance. Mice lacking IRS2:PI3K binding were insulin resistant and severely glucose intolerant due to β-cell failure. In contrast, abrogating the interaction with GRB2 for either IRS1 or IRS2 led to minimal growth and metabolic defects, highlighting the dominant role of PI3K in mediating the physiological effects of IRS proteins.

Our novel knock-in mouse mutants demonstrated effective abrogation of PI3K activity in immunoprecipitates, with evidence of only limited compensation in expression of other IRS proteins in the *Irs1*^YnF^ mice and no evidence for changes in IRS expression in the other mouse lines or the expression of their primary major binding adaptor molecules in all lines. Our GRB2 signalling mutants displayed similar preservation of signalling stoichiometry. Mice with single deletion of the PI3K regulatory subunits p85α and p85β exhibit improved insulin sensitivity and are hypoglycaemic [[63], [64], [65]]. However, these findings are complicated by the fact that gene targeting of specific PI3K subunits can alter the expression of other PI3K subunits, leading to a loss of signalling stoichiometry [42]. In primary hepatocytes of our *Irs1*^YnF^ model, ablation of IRS1:PI3K binding did not affect insulin-stimulated phosphorylation of AKT and ERK, whereas for the IRS2:PI3K mutant, AKT phosphorylation was reduced. Preservation of AKT signalling in *Irs1*^YnF^ mice (and reduced AKT signalling in *Irs2*^YnF^ mice) is consistent with previous studies in mice with liver-specific deletion of *Irs1* or *Irs2* [59] where the authors suggested that reduced PI3K activity in the *Irs1* null liver was compensated for by increased tyrosine phosphorylation of IRS2. Our data is also consistent with the fact that there is no major defect in fasting glucose in *Irs1*^YnF^ mice and no profound metabolic phenotype in the liver of *Irs1* null mice. We did, however, observe reduced AKT signalling in *Irs1*^YnF^ (and *Irs2*^YnF^) MEFs, suggesting there may be cell-type specific effects in terms of downstream signalling depending on any compensation of IRS expression or factors such as the dominant adaptor protein (and their expression) in a particular cell or tissue type.

Compared with the *Irs1* null mouse, our *Irs1*^YnF^ knock-in model also displayed poor viability on a C57BL/6J background, a similar growth restriction from birth, reduced adiposity, increased food intake relative to their size, and insulin resistance, yet exhibited normal glucose handling due to compensatory hyperinsulinaemia from expansion of β-cell mass. Therefore, IRS1-mediated PI3K signalling appears to be the major downstream pathway through which IRS1 regulates growth and metabolism. Similarly, for *Irs2*^YnF^ knock-in vs *Irs2* null mice, specific loss of PI3K binding from IRS2 *in vivo* led to overt diabetes, insulin resistance and reduced β-cell mass, accompanied by obesity and reduced brain size. Overall, both of our PI3K signalling mutant mice largely phenocopy the constitutive null models for both *Irs1* and *Irs2*. Both *Irs1* null mice and our *Irs1*^YnF^ mutants have developmental growth retardation, and it is possible that this contributes to the observed metabolic phenotypes in these animals. However, mice lacking *Irs1* specifically in the liver metabolically phenocopy *Irs1*^YnF^ mice but have normal growth and mice with *Irs1* deletion in muscle or fat also have defective glucose handling and insulin sensitivity while also displaying normal bodyweight [59,66]. These observations suggest that IRS1 signalling plays a key role in metabolic homeostasis in adult animals independent of its effects on growth.

We did, however, observe subtle differences between our PI3K knock-in mice and their global knockout counterparts. These include small differences in tissue-specific insulin sensitivity, in which *Irs1*^YnF^ mice had marked hepatic insulin resistance (but not muscle or adipose tissue), in contrast to the predominantly skeletal muscle insulin resistance previously described in clamp studies of global *Irs1* null mice [32]. It is not precisely clear where these differences stem from, but liver metabolism differs between the fed and fasted states, and mice with liver-specific deletion of *Irs1* exhibit marked insulin resistance upon refeeding with IRS2 proposed to be more important in the fasted state [59]. Differences in signalling (possibly via compensation) in other tissues, as well as developmental effects from constitutive deletion, may underpin the subtle differences observed between *Irs1*^YnF^ vs *Irs1* null mice. Differences in genetic background, environmental conditions or technical differences in the clamp studies could also underlie the divergence in these findings. We also observed unaltered insulin secretion in *Irs1*^YnF^ islets compared with the reduced GSIS found in *Irs1* null islets [61]. This may be due to the previously described compensation in *Irs1* null mice, with upregulation of *Irs2*. These differences could again be explained by subtle differences in genetic background and the effects of the different mutations (*Irs1*^YnF^ vs *Irs1* null) in peripheral tissues, which could indirectly impact islet function. Physiological differences between *Irs2*^YnF^ vs *Irs2* null mice were limited to retained reproductive function in female *Irs2*^YnF^ mice, which was somewhat surprising and suggests a role for non-PI3K pathways in IRS2-mediated reproductive function. Indeed, published evidence suggests that cell-specific loss of MAPK/ERK in the ovary or pituitary gonadotrophs [[67], [68], [69]], but not in GnRH neurons [70], causes profound reproductive failure. Overall, however, our findings show a close phenocopy between *Irs2*^YnF^ mice and their *Irs2* null counterparts. Taken together, the above suggests that PI3K is the major signalling pathway downstream of IRS1 and IRS2, mediating their growth and metabolic effects.

In contrast to our findings in mutant *Irs* mice lacking PI3K binding, ablation of GRB2 binding to either IRS1 or IRS2 (or in combination) did not result in any discernible metabolic or growth-related phenotype. This was surprising, as *in vitro* studies assessing GRB2 binding to IRS1 predicted a major role in recruiting MAPK, and IRS deletion results in multiple growth defects in mice, e.g. somatic growth defects for *Irs1* and tissue-specific growth defects (islets, brain, kidney) in *Irs2*. Our findings are, however, consistent with work by Myers and colleagues [23], who demonstrated that a mutant IRS1 lacking GRB2 binding could still drive mitogenesis *in vitro*. In our knock-in approach, compensatory changes may occur in other IRS proteins that bind to GRB2 in single mutant *Irs1*^Y891F^ or *Irs2*^Y911F^ mice. However, this is unlikely, given that we found no evidence of clear growth or metabolic phenotypes in double *Irs1*^Y891F^/*Irs2*^Y911F^ mice. It remains possible that MAPK signalling is activated via mechanisms outside the canonical GRB2-binding sites on IRS1 (Y891) and IRS2 (Y911). For example, this could occur via alternative direct GRB2 binding sites (Y935 for IRS1 and Y970 for IRS2) or via indirect binding sites identified in studies exploring the phosphotyrosine interactomes of IRS1 and IRS2 [18]. Indeed, Hanke and colleagues demonstrated the breadth of IRS1 and IRS2 interactors and their possible biophysical binding sites (for SHC, SHP2, SHIP2, PLCγ, CSK, CRK, and NCK), some of which mediate insulin signalling *in vitro* [15,24,26,39]. Several of these adaptor proteins have been shown to bind to the same tyrosine residue as PI3K adaptor subunits, albeit indirectly or independent of phosphorylation status. Although challenging to model *in vivo*, we cannot exclude a role for potential additional binders that may have been abrogated using our knock-in approach. However, in our *in vivo* models we observed retention of adaptor binding that was predicted to be abrogated *in vitro*, such as the Y935F mutation on IRS1 with respect to GRB2 binding and the Y734F mutation on IRS2 with respect to SHC binding. Furthermore, MAPK activation has been reported via Ras-PI3K mechanisms [[71], [72], [73]]. However, taken together, the canonical GRB2-binding sites in IRS1 and IRS2 appear to have minimal effects on a range of physiological and growth parameters in mice.

IRS proteins act as molecular scaffolds and integrate a diverse array of downstream signals via their extended, distinct C-terminal regions, which contain multiple phosphorylation sites. The IRS C-terminus is also a target for other modifications, e.g. serine phosphorylation, that appear to play a role in modifying intracellular signalling, both positively and negatively (reviewed extensively in [1]). Our work has focused on tyrosine phosphorylation-dependent mechanisms, but future studies could examine serine phosphorylation events in our knock-in *Irs1*^YnF^ and *Irs2*^YnF^ mice. We note that although our *in vivo* studies may not have revealed all subtle alterations from ablating IRS-dependent PI3K or GRB2 signalling, additional influence from serine phosphorylation is likely to be modulatory, as we observed significant physiological effects from mutating specific tyrosine residues alone that recapitulate almost all constitutive loss of function effects.

Insulin resistance is a key pathophysiological driver and one of the earliest abnormalities in a wide range of human diseases, including T2DM, cardiovascular disease, metabolic liver disease and polyendocrine metabolic ovarian syndrome (PMOS). The pathophysiology of insulin resistance is complex and incompletely understood, partly due to the divergence of intracellular signals downstream of the IR and the influence of both genetic and environmental factors. With respect to IRS proteins and PI3K/AKT activation, functional defects in insulin signalling in the insulin resistant state in skeletal muscle in humans can be mediated by impaired IRS tyrosine phosphorylation, possibly due to the adverse effects of IRS serine phosphorylation [[74], [75], [76]]. Human genetic studies implicate IRS signalling in metabolic disease. For example, GWAS have found *Irs1* to be associated with insulin resistance [77], impaired metabolic profile and reduced adiposity [78], although there is less evidence for *Irs2* in such GWAS studies [79]. However, recent whole-genome sequencing studies found that rare protein-truncating variants (PTVs) in *IRS2* were associated with T2DM [80] and that PTVs in *IRS1* were associated with reduced fat mass and height [80]. Furthermore, human genetic perturbations of the PI3K-AKT pathway with dominant PIK3R1 (p85α, p55α, p50α) mutations and with impaired interaction between IRS1 and p85α cause SHORT syndrome with growth restriction, lipodystrophy, and severe insulin resistance [[81], [82], [83]]. Mice with a knock in allele of the *Pik3r1* R649W missense mutation found in human SHORT syndrome faithfully recapitulate the human condition with protection from dyslipidaemia, hepatic steatosis and reduced adipose function, but not from diabetes, with robust hyperinsulinaemic responses to IR, similar to our *Irs1*^YnF^ mice [[84], [85], [86], [87]]. Combined with our work, these studies suggest that in humans and mice, IRS1 and IRS2 predominantly mediate effects on growth and glucose homeostasis, respectively. Together, this suggests that targeting elements of the IRS signalling machinery may be a therapeutic approach, possibly by enhancing tyrosine phosphorylation (and thereby IRS:PI3K binding) or by preventing (or reversing) inhibitory serine/threonine phosphorylation of IRS.

In summary, we present a novel *in vivo* knock-in approach to assess the metabolic consequences of abrogated PI3K or GRB2 binding to IRS1 or IRS2. Blocking PI3K binding to either IRS1 or IRS2 in mice essentially phenocopied the deletion of that IRS. In contrast, mice unable to recruit GRB2 to either IRS1 or IRS2 showed no discernible changes in growth or metabolism. Overall, our work builds on previous *in vitro* studies of IRS protein-downstream adaptor interactions and highlights the key pathways that are functionally important *in vivo*.

## CRediT authorship contribution statement

**Steven J. Millership:** Writing – review & editing, Writing – original draft, Investigation, Formal analysis, Conceptualization. **Nadia Tyler-Rubinstein:** Writing – review & editing, Investigation, Formal analysis. **Elaine E. Irvine:** Writing – review & editing, Investigation, Formal analysis. **Marika Charalambous:** Writing – review & editing, Investigation, Formal analysis. **Agharul I. Choudhury:** Writing – review & editing, Investigation, Formal analysis. **Santiago Vernia:** Writing – review & editing, Investigation, Formal analysis. **Peter Voshol:** Writing – review & editing, Investigation, Formal analysis. **Antonio Vidal-Puig:** Writing – review & editing, Investigation, Formal analysis. **Lazaros C. Foukas:** Writing – review & editing, Investigation, Formal analysis. **Dominic J. Withers:** Writing – review & editing, Writing – original draft, Supervision, Funding acquisition, Conceptualization.

## Ethics declaration

This study was conducted in accordance with the ARRIVE (Animal Research: Reporting of In Vivo Experiments) guidelines. This study was approved by the Imperial College Animal Welfare and Ethical Review Body and by the UK Home Office.

## Declaration of competing interest

The authors declare that they have no known competing financial interests or personal relationships that could have appeared to influence the work reported in this paper.

## Figures and Tables

**Figure S1:**
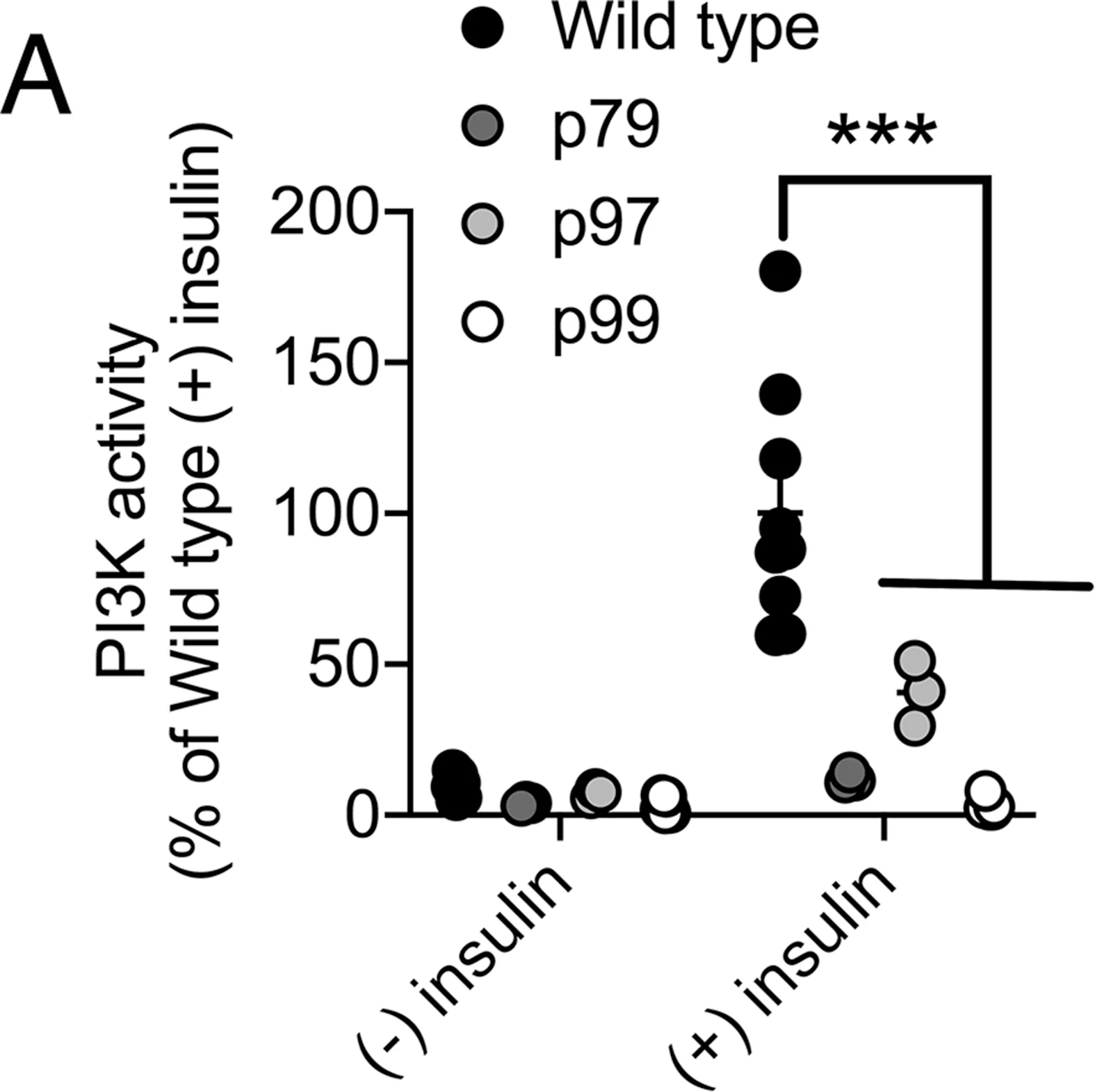
(A) A series of c-Myc-tagged IRS2 mutants with specific tyrosine to phenylalanine conversions as well as a wild type IRS2 control were expressed in CHO-IR cells. Bar graph shows associated PI3K activity in IRS2 immunoprecipitates from insulin-stimulated CHO-IR cells using radiolabelling with [γ^32^P] ATP and subsequent autoradiography (*n* = 3 per mutant vs *n* = 9 for wild type IRS2, ⁎⁎⁎*p* < 0.001).

**Figure S2:**
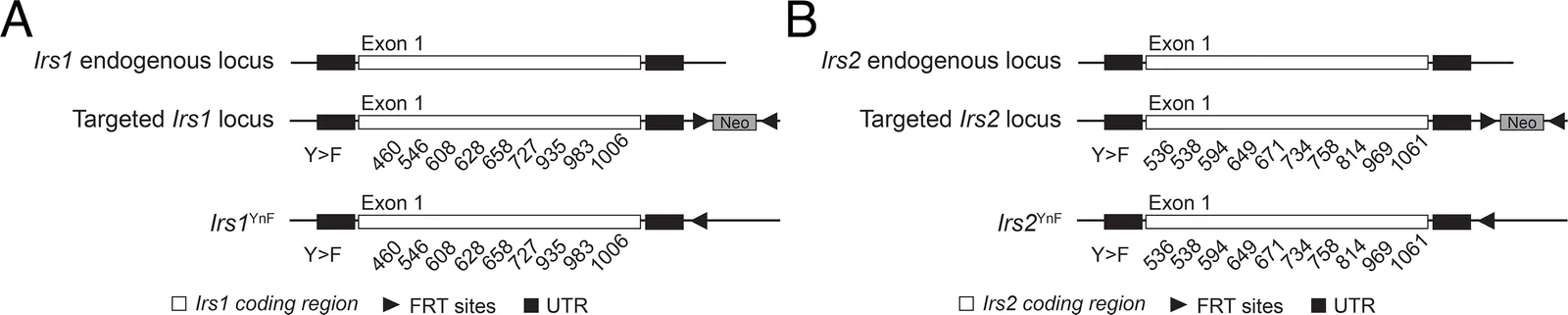
(A, B) Targeted mutation of 9 and 10 tyrosine residues to phenylalanine (Y > F) in exon 1 of *Irs1* and *Irs2* respectively. A *neomycin* cassette (Neo) was flanked by FRT sites and used as a selection marker.

**Figure S3:**
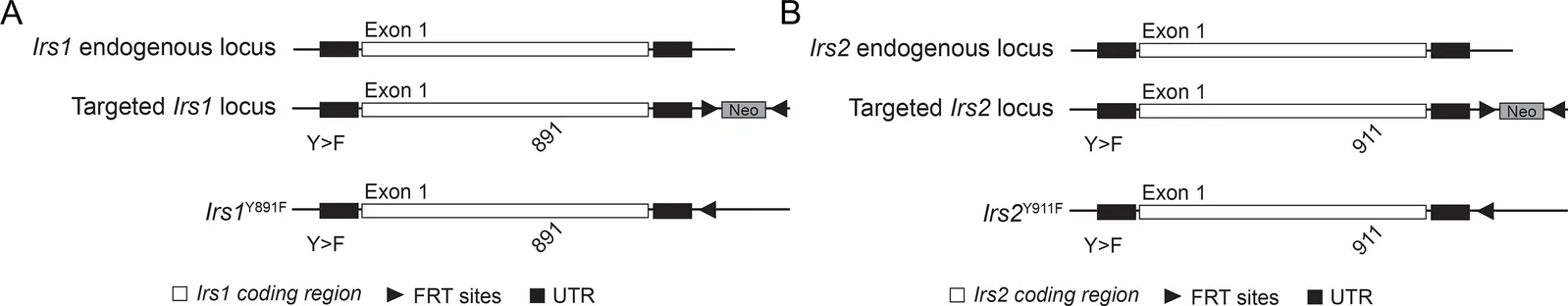
(A, B) Targeted mutation of tyrosine residues to phenylalanine (Y > F) in exon 1 of *Irs1* and *Irs2*, respectively. A *neomycin* cassette (Neo) was flanked by FRT sites and used as a selection marker.

**Figure S4:**
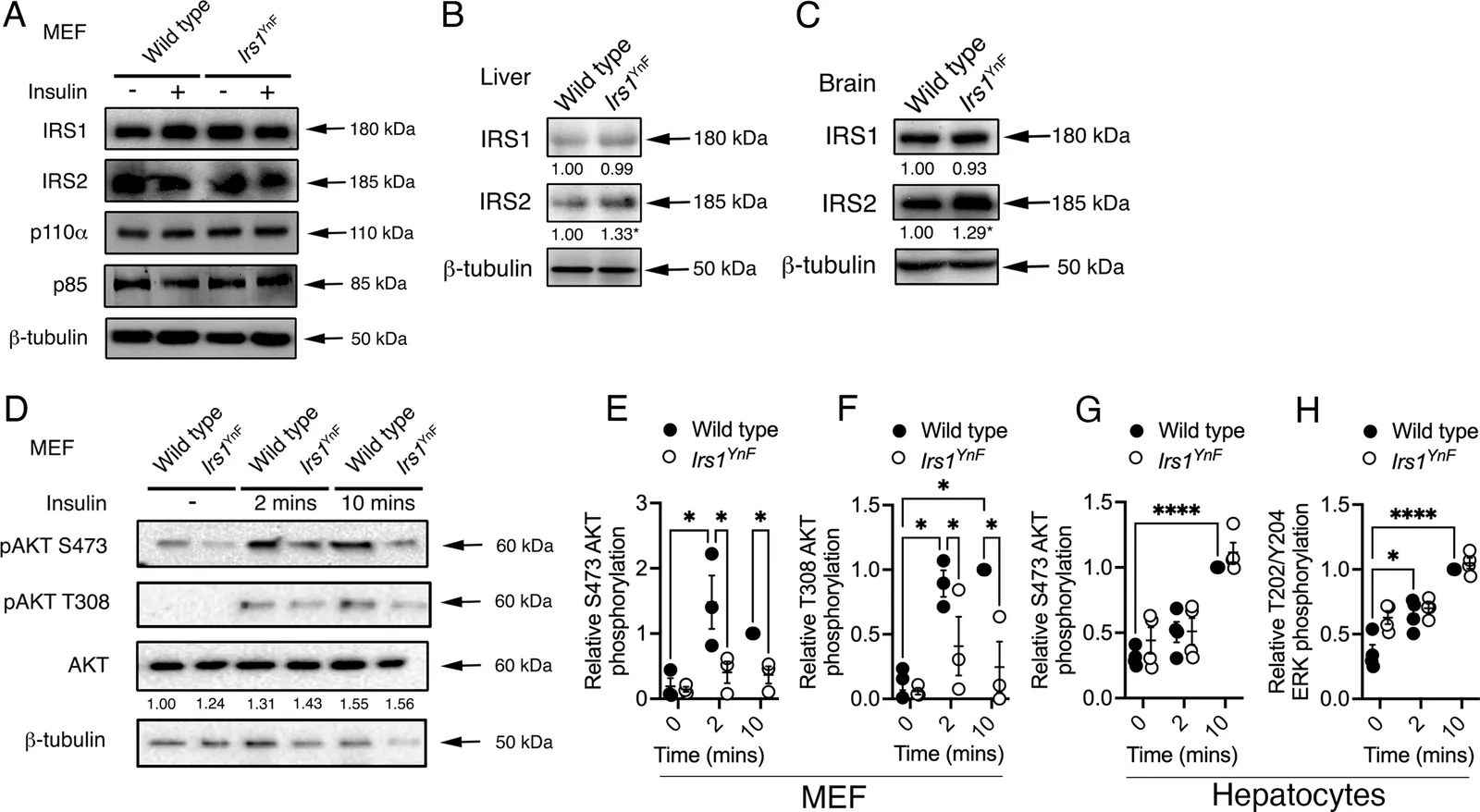
(A-C) Representative Western blotting analysis of protein levels in insulin-treated primary MEFs (A) and liver (B) and brain (C) from *Irs1*^YnF^ and wild type mice (*n* = 4 mice per genotype for MEF and *n* = 8 mice per genotype for liver and brain). β-Tubulin was used as a loading control. Mean band intensities for IRS1 and IRS2 quantified by densitometry from multiple experiments are shown below each panel for (B) and (C), both expressed relative to wild type samples. (D-F) Representative Western blotting analysis of AKT signalling in insulin-treated primary MEFs from *Irs1*^YnF^ and wild type mice (*n* = 3 mice per genotype). β-Tubulin was used as a loading control. Mean band intensities from multiple experiments quantified by densitometry are shown below each panel, all expressed relative to wild type/untreated samples. Associated bar charts in (E) and (F) show ratiometric comparisons between phosphorylated and total forms of AKT, expressed relative to wild type / T = 10 min samples (*n* = 3). (G, H) Ratiometric comparisons between phosphorylated and total forms of AKT and ERK in insulin-treated primary hepatocytes from *Irs1*^YnF^ and wild type mice, expressed relative to wild type / T = 10 min samples (*n* = 4). Data is represented as mean ± SEM (⁎*p* < 0.05, ⁎⁎⁎⁎*p* < 0.0001).

**Figure S5:**
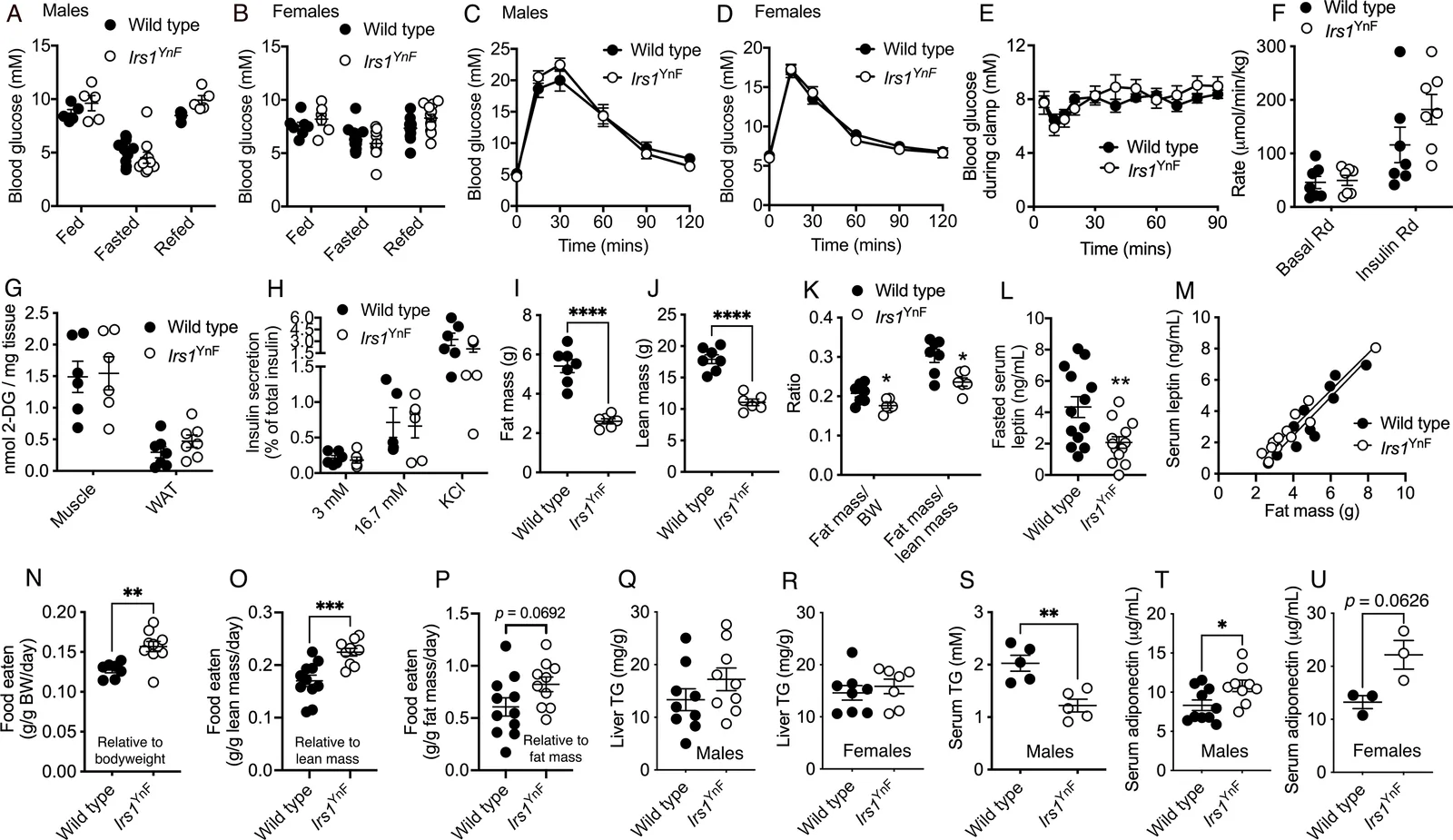
(A, B) Blood glucose levels in adult wild type and *Irs1*^YnF^ mice in both the fed, fasted and refed state (males: *n* = 5, *n* = 10 and *n* = 5 mice per genotype for fed, fasted and refed respectively; females: *n* = 5–6, *n* = 11–14 and *n* = 12 mice per genotype for fed, fasted and refed respectively). (C, D) Glucose tolerance testing in adult wild type and *Irs1*^YnF^ mice (*n* = 9 and *n* = 10 per genotype for males and females respectively). (E) Blood glucose levels in adult female wild type and *Irs1*^YnF^ mice during hyperinsulinaemic-euglycaemic clamps (*n* = 8 and *n* = 11 for wild type and *Irs1*^YnF^ respectively). (F) Rates of basal and hyperinsulinaemic-euglycaemic (insulin) glucose disposal (Rd) in adult female wild type and *Irs1*^YnF^ mice (*n* = 7 per genotype). (G) Uptake of ^14^C-2-deoxyglucose (2-DG) into muscle and white adipose tissue (WAT) in adult female wild type and *Irs1*^YnF^ mice (*n* = 6–9 and *n* = 8–11 per genotype for muscle and WAT, respectively). (H) Insulin secretion in static incubations of primary isolated islets from adult male wild type and *Irs1*^YnF^ mice was determined *in vitro* under low glucose (3 mM), high glucose (16.7 mM) and KCl-stimulated conditions. Insulin secretion into the media was expressed as a percentage of total cellular insulin. (I–K) Fat mass (I), lean mass (J) and adiposity as a ratio of bodyweight (BW) and fat mass vs lean mass (K) in female wild type and *Irs1*^YnF^ mice as quantified by MRI scanning (*n* = 7 and 6 for wild type and *Irs1*^YnF^ respectively). (L) Fasted serum leptin levels in adult female wild type and *Irs1*^YnF^ mice (*n* = 13 per genotype). (M) Linear regression analysis of fat mass vs serum leptin in adult female wild type and *Irs1*^YnF^ mice (*n* = 11 and *n* = 14 for wild type and *Irs1*^YnF^ respectively). *R*^2^ value (coefficient of determination): wild type *R*^2^ = 0.83⁎⁎⁎; *Irs1*^YnF^*R*^2^ = 0.93⁎⁎⁎; wild type vs *Irs1*^YnF^ slope: *p* = 0.82. (N–P) Food intake in adult male wild type and *Irs1*^YnF^ mice vs bodyweight (N) (*n* = 7 and *n* = 10 for wild type and *Irs1*^YnF^ respectively), lean mass (O) and fat mass (P) (*n* = 11 and *n* = 10 for wild type and *Irs1*^YnF^ respectively). (Q, R) Fasted liver triglyceride (TG) levels in adult wild type and *Irs1*^YnF^ mice (*n* = 9 per genotype for males, *n* = 8 and *n* = 7 for wild type and *Irs1*^YnF^ respectively for females). (S) Fasted serum triglyceride (TAG) levels in adult male wild type and *Irs1*^YnF^ mice (*n* = 5 per genotype). (T, U) Fasted serum adiponectin levels in adult wild type and *Irs1*^YnF^ mice (*n* = 10 and *n* = 9 for wild type and *Irs1*^YnF^ for males, respectively; *n* = 3 per genotype for females). In all panels, data are represented as mean ± SEM (⁎*p* < 0.05, ⁎⁎*p* < 0.01, ⁎⁎⁎*p* < 0.001, ⁎⁎⁎⁎*p* < 0.0001).

**Figure S6:**
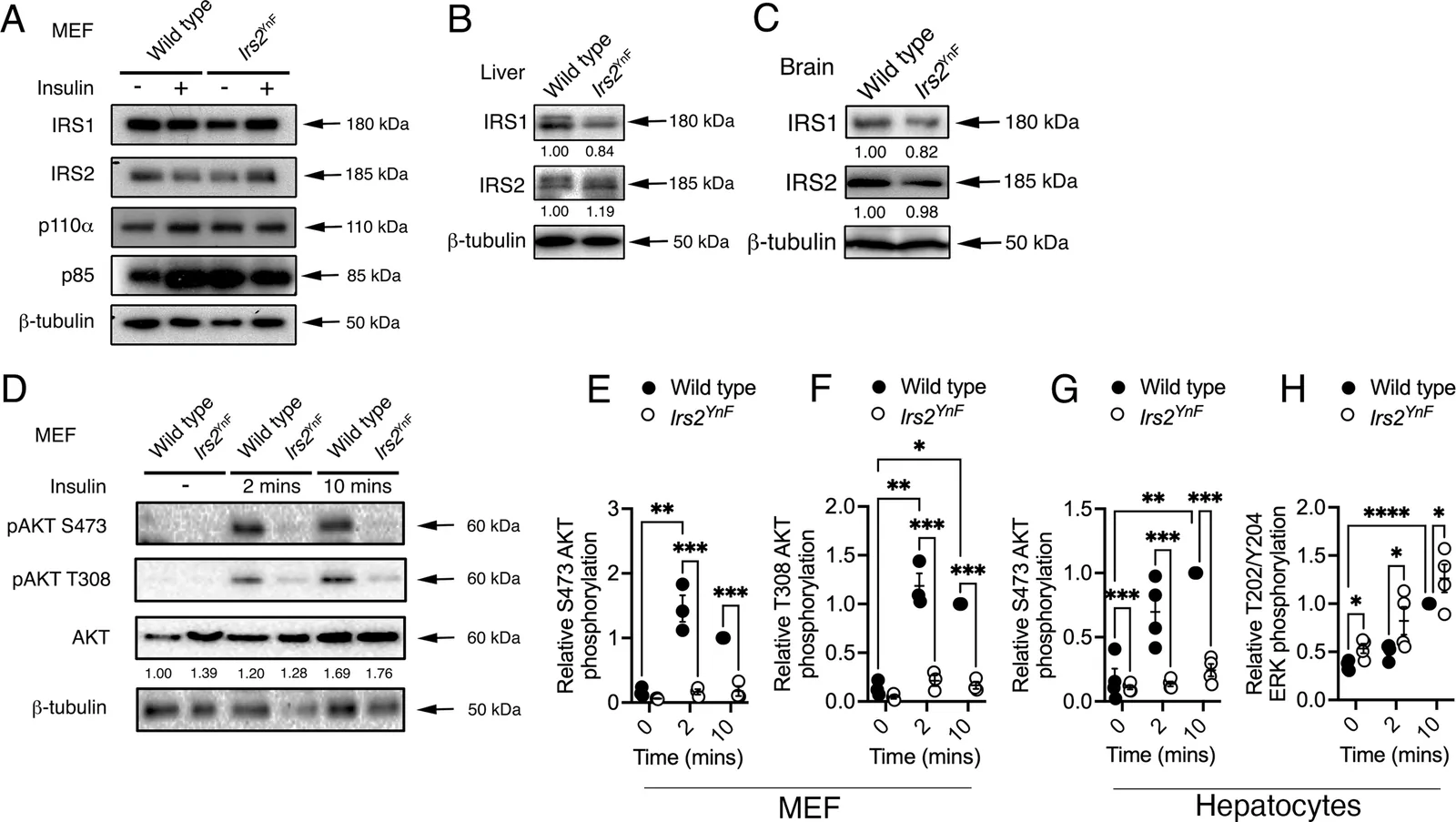
(A-C) Representative Western blotting analysis of protein levels in insulin-treated primary MEFs (A) and liver (B) and brain (C) from *Irs2*^YnF^ and wild type mice (*n* = 4 mice per genotype for MEF and *n* = 8 mice per genotype for liver and brain). β-Tubulin was used as a loading control. Mean band intensities for IRS1 and IRS2 quantified by densitometry from multiple experiments are shown below each panel for (B) and (C), both expressed relative to wild type samples. (D-F) Representative Western blotting analysis of AKT signalling in insulin-treated primary MEFs from *Irs2*^YnF^ and wild type mice (*n* = 3 mice per genotype). β-Tubulin was used as a loading control. Mean band intensities from multiple experiments quantified by densitometry are shown below each panel, all expressed relative to wild type/untreated samples. Associated bar charts in (E) and (F) show ratiometric comparisons between phosphorylated and total forms of AKT, expressed relative to wild type / T = 10 min samples (*n* = 3). (G, H) Ratiometric comparisons between phosphorylated and total forms of AKT and ERK in insulin-treated primary hepatocytes from *Irs2*^YnF^ and wild type mice, expressed relative to wild type / T = 10 min samples (*n* = 4). Data is represented as mean ± SEM (⁎*p* < 0.05, ⁎⁎*p* < 0.01, ⁎⁎⁎*p* < 0.001, ⁎⁎⁎⁎*p* < 0.0001).

**Figure S7:**
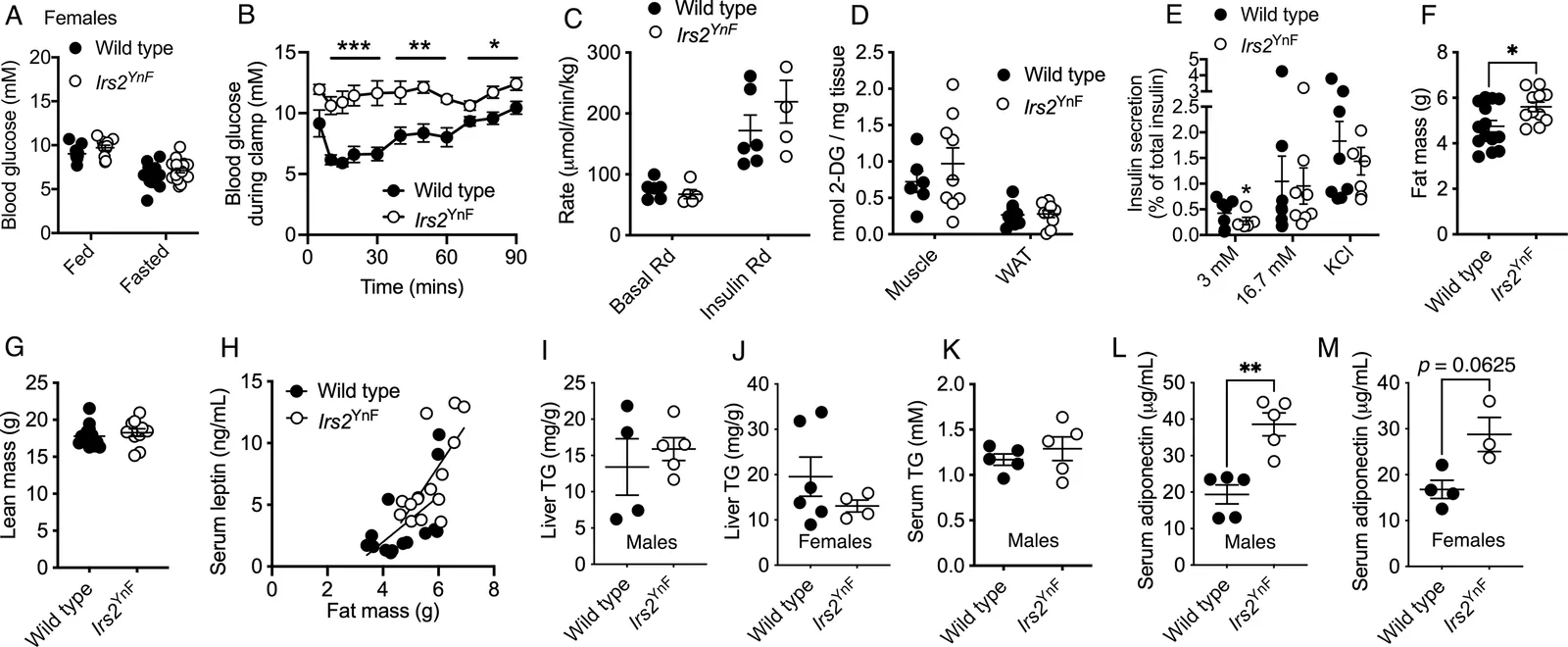
(A) Blood glucose levels in adult 10 week old female wild type and *Irs2*^YnF^ mice in both the fed and fasted state (*n* = 11 and *n* = 18 mice per genotype for fed and fasted respectively). (B) Blood glucose levels in adult female wild type and *Irs2*^YnF^ mice during hyperinsulinaemic-euglycaemic clamps (*n* = 6 per genotype). (C) Rates of basal and hyperinsulinaemic-euglycaemic (insulin) glucose disposal (Rd) in adult female wild type and *Irs2*^YnF^ mice (*n* = 6 and *n* = 5 for wild type and *Irs2*^YnF^ respectively). (D) Uptake of ^14^C-2-deoxyglucose (2-DG) into muscle and white adipose tissue (WAT) in adult female wild type and *Irs2*^YnF^ mice (*n* = 6–9 and *n* = 8–11 per genotype for muscle and WAT, respectively). (E) Insulin secretion in static incubations of primary isolated islets from adult male wild type and *Irs2*^YnF^ mice was determined *in vitro* under low glucose (3 mM), high glucose (16.7 mM) and KCl-stimulated conditions. Insulin secretion into the media was expressed as a percentage of total cellular insulin. (F, G) Fat mass (F) and lean mass (G) in female wild type and *Irs2*^YnF^ mice as quantified by MRI scanning (*n* = 15 and 11 for wild type and *Irs1*^YnF^ respectively. (H) Linear regression analysis of fat mass vs serum leptin in adult female wild type and *Irs2*^YnF^ mice (*n* = 14 and *n* = 15 for wild type and *Irs2*^YnF^ respectively). *R*^2^ value (coefficient of determination): wild type *R*^2^ = 0.35⁎; *Irs2*^YnF^*R*^2^ = 0.48⁎⁎; wild type vs *Irs2*^YnF^ slope: *p* = 0.22. (I, J) Fasted liver triglyceride (TG) levels in adult wild type and *Irs2*^YnF^ mice (*n* = 4 and *n* = 5 for wild type and *Irs2*^YnF^ respectively for males; *n* = 6 and *n* = 4 for wild type and *Irs2*^YnF^ respectively for females). (K) Fasted serum triglyceride (TAG) levels in adult male wild type and *Irs2*^YnF^ mice (*n* = 5 per genotype). (L, M) Fasted serum adiponectin levels in adult wild type and *Irs2*^YnF^ mice (*n* = 5 per genotype for males; *n* = 4 and *n* = 3 for wild type and *Irs2*^YnF^ respectively for females). In all panels, data are represented as mean ± SEM (⁎*p* < 0.05, ⁎⁎*p* < 0.01, ⁎⁎⁎*p* < 0.001).

**Figure S8:**
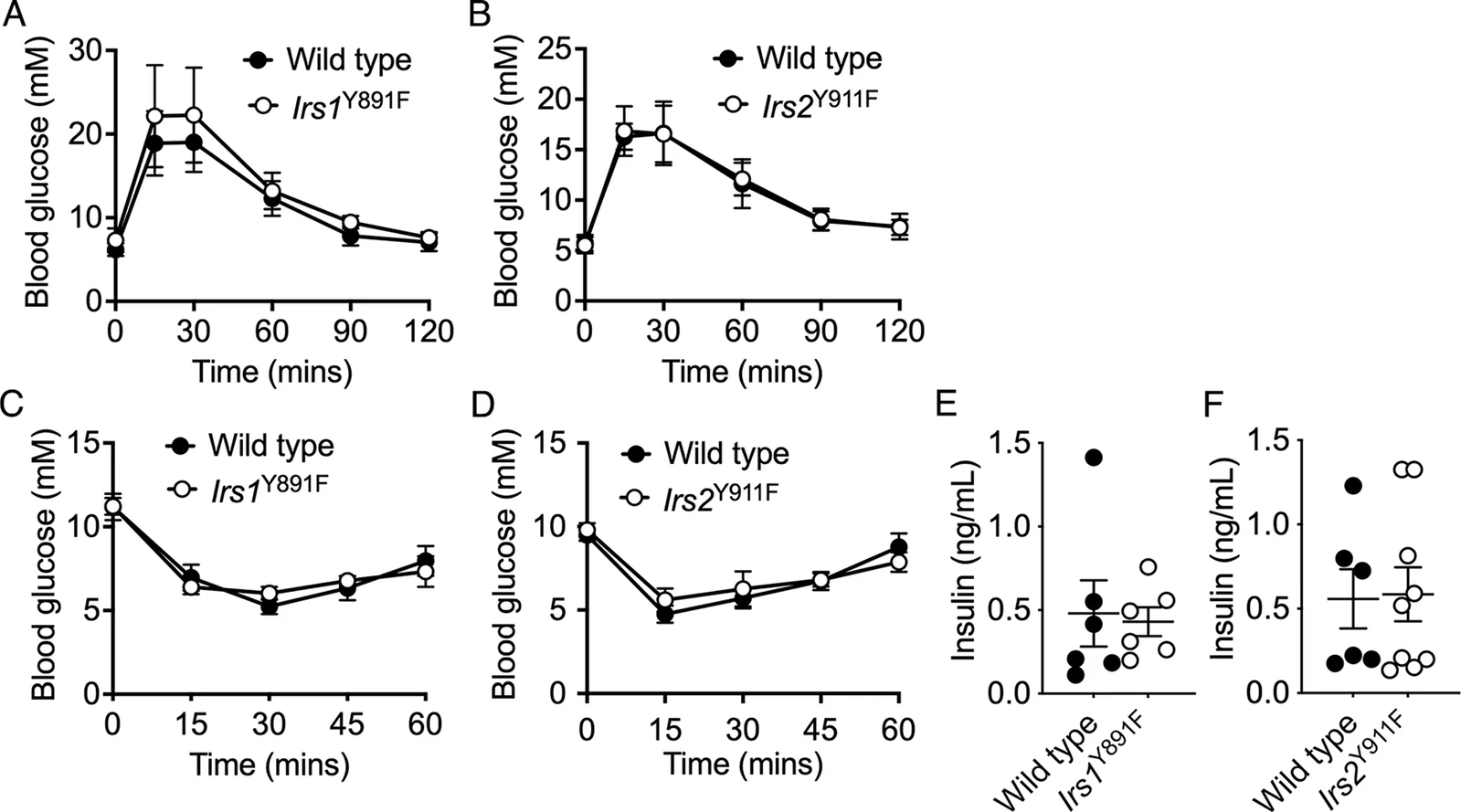
Physiological analysis of male wild type and *Irs1*^Y891F^ mice and male wild type and *Irs2*^Y911F^ mice including glucose tolerance (A, B) (*n* = 6 per genotype for *Irs1*^Y891F^; *n* = 9 for *Irs2*^Y911F^ vs *n* = 6 for wild type controls), insulin tolerance (C, D) (*n* = 5 for *Irs1*^Y891F^ vs *n* = 6 for wild type controls; *n* = 8 for *Irs2*^Y911F^ vs *n* = 9 for wild type controls) and fasted serum insulin levels (E, F) (*n* = 6 per genotype for *Irs1*^Y891F^; *n* = 9 for *Irs2*^Y911F^ vs *n* = 6 for wild type controls). In all panels, data are represented as mean ± SEM.

**Figure S9:**
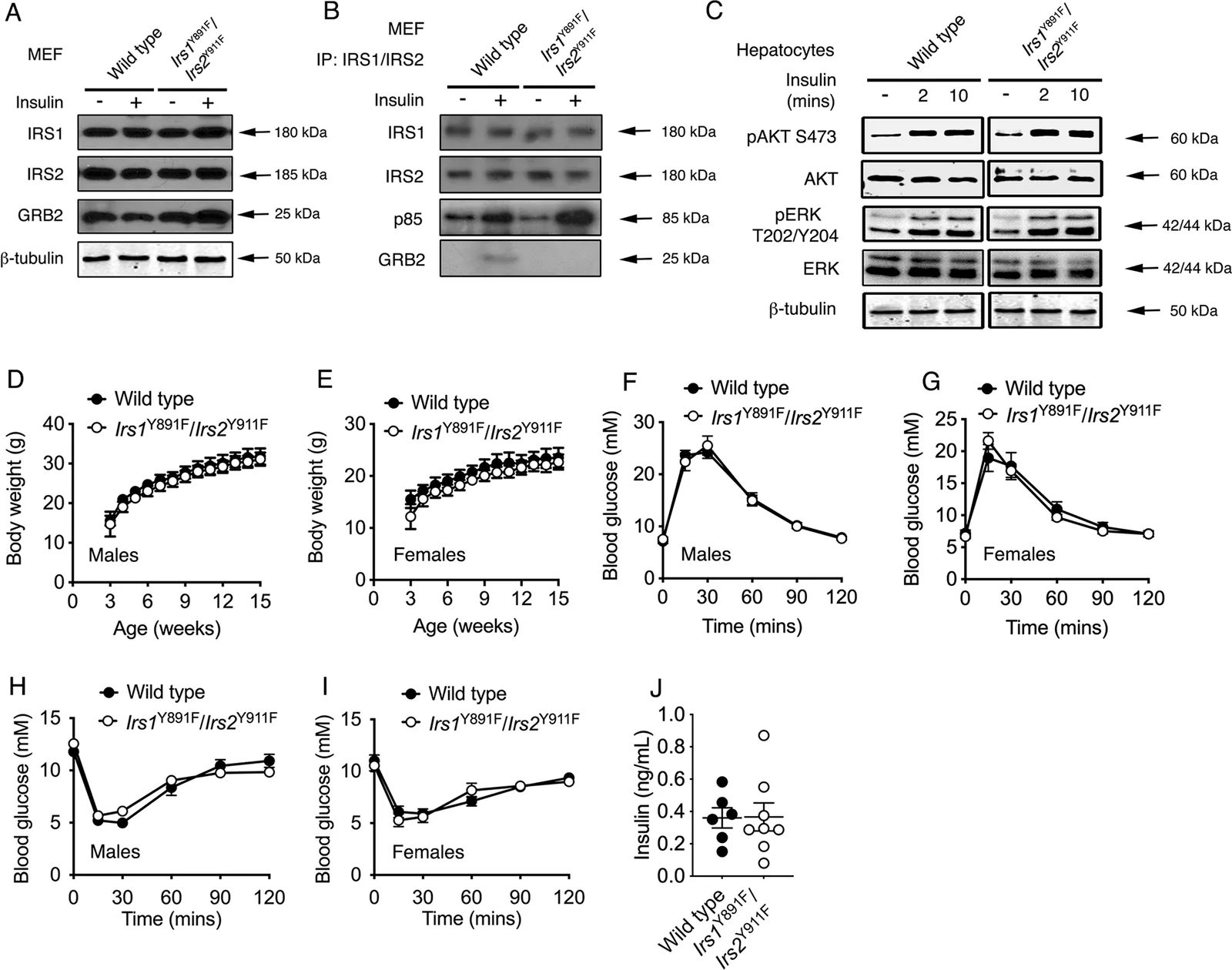
(A, B) Lysates (inputs shown in A) from *Irs1*^Y891F^/*Irs2*^Y911F^ insulin-treated MEFs immunoprecipitated using anti-IRS1 and anti-IRS2 antibodies. Proteins in IPs were detected by Western blotting using antibodies against IRS1, IRS2, p85 and GRB2 (B). The panel shows a representative blot from three independent experiments. (C) Representative Western blotting analysis of protein levels in insulin-treated primary hepatocytes from *Irs1*^Y891F^/*Irs2*^Y911F^ and wild type mice (*n* = 3 mice per genotype). β-Tubulin was used as a loading control. (D-J) Physiological analysis of male and female wild type and double mutant *Irs1*^Y891F^/*Irs2*^Y911F^ mice including body weight (D, E, (males: *n* = 8 and *n* = 11 for wild type and *Irs1*^Y891F^/*Irs2*^Y911F^, respectively; females: *n* = 6 and *n* = 7 for wild type and *Irs1*^Y891F^/*Irs2*^Y911F^, respectively), glucose tolerance (F, G) and insulin tolerance (H, I) (males: *n* = 6 and *n* = 8 for wild type and *Irs1*^Y891F^/*Irs2*^Y911F^, respectively; females: *n* = 6 and *n* = 7 for wild type and *Irs1*^Y891F^/*Irs2*^Y911F^, respectively) and fasted serum insulin levels in male mice (J), (*n* = 6 and *n* = 8 for wild type and *Irs1*^Y891F^/*Irs2*^Y911F^, respectively). In all panels, data are represented as mean ± SEM (⁎*p* < 0.05).

## Data Availability

Data will be made available on request.
