# Supplementary material for: PI3K is the dominant signalling pathway recruited by IRS proteins to mediate their effects on metabolism and growth
Source: Mol Metab. 2026 Oct 6;114:102450. doi: 10.1016/j.molmet.2026.102450 (PMC13694759; doi:10.1016/j.molmet.2026.102450)
Supplement: Supplementary file 1 [file mmc1.docx]

| Supplemental Table 1. List of primary antibodies used for Western blotting | | |
| --- | --- | --- |
| Antibody | Clone | Source |
| IRS1 | 4.2.2 | Merck Millipore (05-1085) |
| IRS2 | 9.5.2 | Merck Millipore (MABS15) |
| IRS1 | - | In house |
| IRS2 | - | In house |
| S473 phosphorylated AKT | - | Cell Signalling (#9271) |
| T308 phosphorylated AKT | - | Cell Signalling (#9275) |
| Pan (total) AKT | 40D4 | Cell Signalling (#2920) |
| SHC | - | BD Biosciences (610081) |
| p85 | - | Merck Millipore (06-195) |
| p110α | - | Cell Signalling (#4255) |
| GRB2 | M331 | Abcam (ab86713) |
| T202 / Y204 phosphorylated ERK | - | Cell Signalling (#9101) |
| Pan (total) ERK | - | Cell Signalling (#9102) |
| β-tubulin | 9F3 | Cell Signalling (#2128) |
